# Materials genomics methods for high-throughput construction of COFs and targeted synthesis

**DOI:** 10.1038/s41467-018-07720-x

**Published:** 2018-12-10

**Authors:** Youshi Lan, Xianghao Han, Minman Tong, Hongliang Huang, Qingyuan Yang, Dahuan Liu, Xin Zhao, Chongli Zhong

**Affiliations:** 1 State Key Laboratory of Organic-Inorganic Composites, Beijing University of Chemical Technology, Beijing, 100029 China; 2 Key Laboratory of Synthetic and Self-Assembly Chemistry for Organic Functional Molecules, Center for Excellence in Molecular Synthesis, Shanghai Institute of Organic Chemistry, Chinese Academy of Sciences, Shanghai, 200032 China; 3 School of Chemistry and Materials Science, Jiangsu Normal University, Xuzhou, 221116 China; 4 State Key Laboratory of Separation Membranes and Membrane Processes, Tianjin Polytechnic University, Tianjin, 300387 China

## Abstract

Materials genomics represents a research mode for materials development, for which reliable methods for efficient materials construction are essential. Here we present a methodology for high-throughput construction of covalent organic frameworks (COFs) based on materials genomics strategy, in which a gene partition method of genetic structural units (GSUs) with reactive sites and quasi-reactive assembly algorithms (QReaxAA) for structure generation were proposed by mimicking the natural growth processes of COFs, leading to a library of 130 GSUs and a database of ~470,000 materials containing structures with 10 unreported topologies as well as the existing COFs. As a proof-of-concept example, two generated 3D-COFs with **ffc** topology and two 2D-COFs with existing topologies were successfully synthesized. This work not only presents useful genomics methods for developing COFs and largely extended the COF structures, but also will stimulate the switch of materials development mode from trial-and-error to theoretical prediction-experimental validation.

## Abstract (web summary)

The discovery of new covalent organic framework (COF) topologies is often led by trial-and-error experiments. Here, the authors present a methodology for high throughput construction of COFs based on a materials genomics strategy and demonstrate the synthesis of the generated 2D and 3D-COFs.

## Introduction

Advanced nanoporous materials are of great importance in various fields. Nowadays, materials discovery is primarily based on scientific intuition and expensive trial-and-error experimentation^1^. Such traditional approaches often result in lengthy time from initial research to practical application. In recognition of this, the United States in 2011 announced the Materials Genome Initiative (MGI) to accelerate advanced materials innovation, one of the main issues is to develop high-throughput computational tools for materials design to construct large materials database^1–4^. The subsequent programs with same ambition of Horizon 2020 and Chinese-version MGI were launched simultaneously in Europe and China. In the MGI, one of the significant challenges is to computationally construct vast space of possible materials for identifying optimal candidates. In this aspect, several pioneering studies have been reported in the literature^5,6^. An excellent example is the contribution of Snurr and coworkers^7^ to metal-organic frameworks (MOFs) that consist of organic linkers connected via metal ions^8,9^. They generated an impressive database of over 130,000 hypothetical materials using recursive, geometry-based algorithms to recombine a set of building blocks compiled from known MOFs. Alternative topology-based approaches were also developed in a top-down manner to construct massive hypothetical structures using underlying nets^10–12^. In addition to these hypothetical compounds, large archives of experimental MOFs were also built from the crystal data deposited in the Cambridge Structural Database (CSD)^13–15^.

Covalent organic frameworks (COFs) represent another emerging class of nanoporous crystalline materials distinguished from MOFs, which are assembled from organic reactants via covalent bonds^16,17^. Depending on the structures of building units, COFs can be formed into those with either two- (2D) or three-dimensional (3D) structures^18–20^. In contrast to MOFs, the structures of COFs reported experimentally are very limited (~320)_,_ which is also the case in computational studies^21–23^. Apart from collection of experimental COFs^24^, a relatively large database was achieved by Martin et al.^25^, which contains 4147 hypothetical materials via framework interpenetration of 620 unique 3D-COFs assembled from their topology-based constructor. Recently, this method was further adopted by Smit and coworkers^26^ to greatly expand COF database. Since chemistry of COFs can be theoretically very rich, leading to these progresses still far from the need of computational screening from the viewpoint of the MGI. Furthermore, there is no large database available so far for 2D-COFs, although such materials have become a research hotspot in many areas^27^.

Therefore, in complement with the library of nanoporous materials containing MOFs, our idea in this work is to develop relevant construction methods for COFs, particularly for 2D-COFs using a self-adaption algorithm to determine interlayer spacing. A database of ~470,000 COFs is built, from which four COFs are targeted synthesized as a proof-of-concept example, demonstrating the applicability of our materials construction methods. This work not only presents useful methods and a large COF database, but also gives an example for developing advanced materials based on materials genomics strategy.

## Results

### Partition method for COF genes and their library

**Fig. 1:**
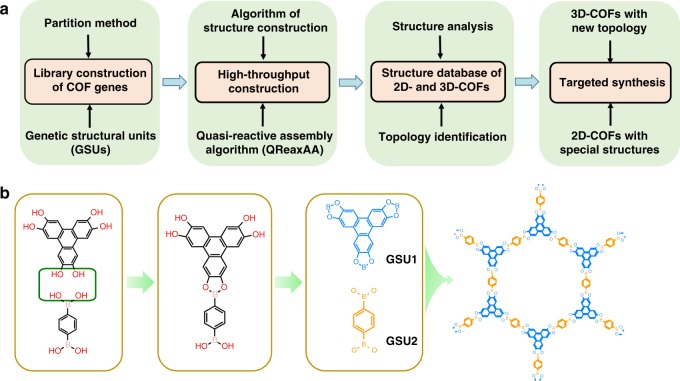
General idea and partition method for COF genes. **a** Schematic illustration of the proposed materials genomics strategy. **b** A concept of genetic structural units with reactive sites was proposed for COF gene partition by mimicking the chemical synthesis processes of COFs, where a dehydration reaction of boronic acid and diol is taken as an example for illustration

The idea of this work is shown in Fig. 1a, in which the first step is to build the library of COF genes for structure construction. Different from the synthesis of MOFs usually using organic ligands and metal salts, COFs are generally synthesized via polycondensation reactions of organic monomers (or molecules) and their original architectures are principally maintained in the resultant structures. Considering this, we proposed a gene partition method namely genetic structural units (GSUs), which are the structural units with reactive sites derived by mimicking COFs natural growth process, and thus have heredity (Fig. 1b). Such treatment enables a rapid collection of the GSUs from existing materials and rational design using chemical knowledge. In parallel, the partition method was constrained to follow three rules: retaining the reactive sites of GSUs as far as possible at reaction terminals of reactants occurring in specific reaction processes; keeping the GSUs as relatively-integral usual molecules to the greatest extent; conveniently defining connection sites on the final GSUs for subsequent structural construction. Starting from COF structures that could be potentially synthesized from common reaction types (such as Boron-, Triazine-, Imine-, Boron/Imine-based ones)^17–19^, our partition method results in a total of 130 GSUs in the gene library of COFs (the library of GSUs is available from the authors as well as on the website: https://figshare.com/s/f8377d33bb9e9a73935a), possessing a variety of geometries, numbers of reactive sites and chemical compositions. Owing to the organic nature of COFs, these GSUs are further categorized into three types: center, linker and functional group) (see the details in Supplementary Fig. 1).

### Materials genomics-based algorithms for COF construction

Besides the definition of materials genes, efficient computational tool for rapidly assembling COF structures on a large scale is another technical key in the viewpoints of materials genomics. Our genomic procedure shares much in spirit with the method of Wilmer et al.^7^ for MOF construction, but with distinct differences in consideration of the features of COFs as well as the efficiency of construction process, and a high-throughput construction method called quasi-reactive assembly algorithms (QReaxAA) was proposed. Firstly, to conveniently generate a variety of COFs, we adopted three different geometrical positioning methods to connect the reactive sites predefined on the GSUs, as shown in Supplementary Fig. 2. Secondly, our strategy is to directly explore the possibility of every combination of the GSUs, avoiding defining all possible arrangements with enumerable strings that are applied in their procedure. Thirdly, parent structures of COFs are generated in our procedure prior to chemical modifications. Compared with the procedure of attempting to assemble structures using three groups of building blocks simultaneously^7^, such post-functionalization operation in our QReaxAA on constructible parent frameworks can greatly increase the efficiency of high-throughput computational materials design. Finally, for large-scale construction of 2D-COFs with layered structures, special attention needs to be paid on how to appropriately arrange interlayer spacing. To address this issue, a self-adaption algorithm was proposed in this work. The general process of framework construction is given as follows.

**Fig. 2:**
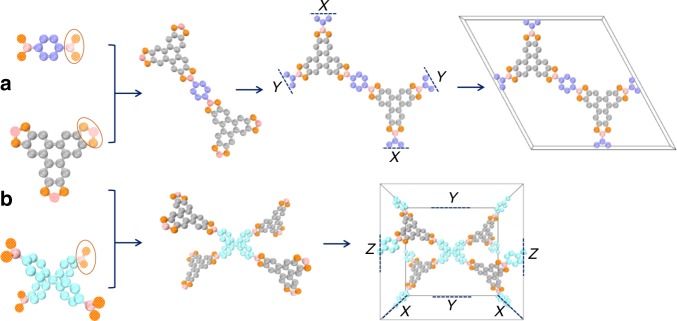
Processes for 2D- and 3D-COF constructions using the method of QReaxAA. **a** The two GSUs on the left can be combined stepwise to construct a 2D-COF framework. When finding respective repetitive connectivity in *X* and *Y* directions, a periodic boundary is imposed on each direction to connect the GSUs located at both sides. The overall structure is formed by applying the third periodic boundary using the interlayer spacing arranged from our self-adaption algorithm. **b** The two GSUs can be combined to generate a 3D-COF framework. The combination step is similar to that of 2D-COFs but with three periodicities need to be found

Parent structures of COFs are generated by stepwise connecting center- and linker-type of GSUs. The so-assembled frameworks are only allowed to contain one center-kind and at most two linker-kinds of GSUs. Once a success is achieved, chemical modification will be performed by attempting all kinds of GSUs categorized as functional groups. For 3D-COFs, both our approach and the approach by Wilmer et al. are straightforward, as shown in Fig. 2. As for 2D-COFs, their interlayer spacing can be approximately arranged using our self-adaption algorithm. More specifically, by examining the structures of existing 2D-COFs, we empirically deduce the contributions of each center- and linker-kind of GSUs to interlayer spacing (see more detailed descriptions in the Methods section and Supplementary Fig. 5).

### Library of the generated COFs

**Fig. 3:**
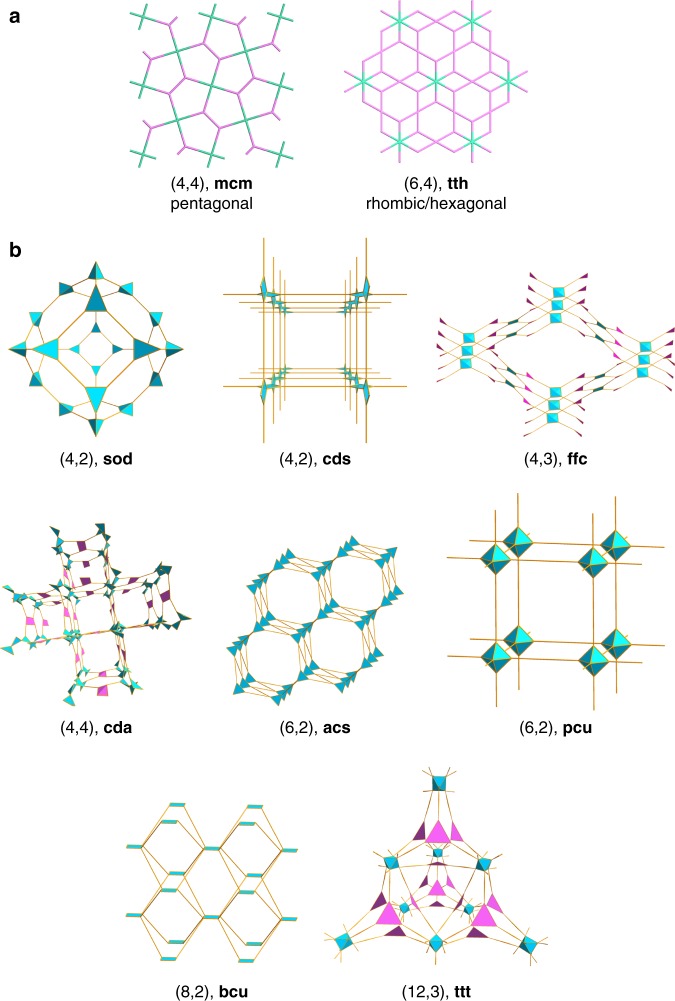
Unreported topologies for the designed COFs. **a** 2D-COFs. **b** 3D-COFs

To check the validity of our genomic approach, we compared the experimentally reported COF structures with our generated ones and their energetically-relaxed counterparts by molecular mechanics (MM) optimization. For this purpose, six 2D-COFs and four 3D-COFs were taken as examples and the comparison metrics are their structural features which include cell parameters, surface area and void fraction, etc. Supplementary Tables 2 and 3 show good agreement between these descriptors for each material and it is also the case for the generated structures composed of various GSUs (Supplementary Table 4), demonstrating our genomic construction methods are capable of effectively generating COFs on a large scale. A total of 471,990 structures (166,684 2D-COFs and 305,306 3D-COFs) were generated in our library; apart from covering the experimental COFs reported so far (319), the library contains 471,671 generated COFs in which 10 unreported topologies were identified; this enriches the COF topologies and structures largely. All the topologies contained in the library are given in Table 1 and Fig. 3 (also Supplementary Fig. 6). Our COF database can play a great role in complementary to that recently reported by Mercado et al.^26^, which contains 61,199 3D- and 8,641 2D-COFs. It should be pointed out that our database at the moment only contains the structures featuring no mutually interdigitated frameworks.

**Table 1:** Structural information of the existing and designed COFs in our database

Categories of COFs		Combination way^a^	Linkage^b^	Topology	Example^c^
2D	Existing COFs	*C*_3_ + *C*_2,_ *C*_2_ + *C*_2_	(3,2)	**hcb**	COF-LZU1^36^, COF-1^34^
		*C*_3_ + *C*_3_	(3,3)	**hcb**	COF-6^35^
		*C*_4_ + *C*_2_	(4,2)	**sql, kgm, fxt**	Pc-PBBA-COF^43^, 4PE-1P^44^, TP-COF-BZ^45^
		*C*_4_ + *C*_4_	(4,4)	**sql, bex, fes**	POR-COF^46^, SIOC-COF-5^47^, ICOF-1^48^
		*C*_6_ + *C*_2_	(6,2)	**hxl**	HPB-COF^49^
		*C*_6_ + *C*_3_	(6,3)	**kgd**	HAT-NTBA-COF^50^
	Designed COFs	*C*_4_ + *C*_4_	(4,4)	**mcm**	GCOF-BP
		*C*_6_ + *C*_4_	(6,4)	**tth**	GCOF-HT
3D	Existing COFs	*C*_3_ + *C*_3_	(3,3)	**srs**	SiCOF-5^51^
		*C*_4_ + *C*_3_	(4,3)	**ctn, bor**	COF-102^52^, COF-108^52^
		*C*_4_ + *C*_2_	(4,2)	**dia**	COF-300^30^
		*C*_4_ + *C*_4_	(4,4)	**pts**	3D-Py-COF^53^
		*C*_12_ + *C*_4_	(12,4)	**rra**	CD-COFs^54^
	Designed COFs	*C*_4_ + *C*_2_	(4,2)	**sod**	GCOF-MT
		*C*_4_ + *C*_2_	(4,2)	**cds**	GCOF-SB
		*C*_4_ + *C*_3_	(4,3)	**ffc**	3D-ETTA-TFPA
		*C*_6_ + *C*_2_	(6,2)	**pcu**	GCOF-HB
		*C*_4_ + *C*_4_	(4,4)	**cda**	GCOF-ST
		*C*_6_ + *C*_2_	(6,2)	**acs**	GCOF-TT
		*C*_8_ + *C*_2_	(8,2)	**bcu**	GCOF-OD
		*C*_12_ + *C*_3_	(12,3)	**ttt**	GCOF-DH

^a^Possible combination ways of reaction substrates, where C_*n*_ means the substrates with *n* reactive terminals

^b^The linkage can be inferred from the combination way of reaction substrates. For instance, the (3,2) linkage can be realized by the co-condensation of *C*_3_ + *C*_2_ or the self-condensation of *C*_2_

^c^The representatives of the designed COFs are shown in Supplementary Fig. 7

The topologies identified for both 2D- and 3D-COFs are summarized as follows. For 2D-COFs, the **mcm** one is achieved by applying an existing (4,4) linkage to build structures, and the **tth** topology with two types of pores is realized via (6,4) linkage (Fig. 3a). For 3D-COFs, a total of 8 topologies are constructed **(**Fig. 3b): applying (6,2), (8,2) and (12,3) linkages results in topologies of **pcu**, **acs**, **bcu** and **ttt**, while using the known (4,2) and (4,3) linkages respectively leads to the **sod** and **ffc** topologies. In addition, the self-periodic extensions of the (4,2) and (4,4) linkages respectively generate structures with **cds** and **cda** topologies that possess continuous chains of center-type GSUs.

Some representatives of the generated structures with above topologies and the Bio-COFs generated using biologically-compatible GSUs are shown in Supplementary Figs. 7 and 8, respectively. The generated COF database encompasses a large number of COFs with distinctive structures, and the associated structural properties span a wide range of values (Supplementary Fig. 9), ensuring a plentiful and useful reservoir complementary to the existing library of nanoporous materials. This database is available from the authors as well as on the website: https://figshare.com/s/c7e3b7610a71b9d64210.

### Targeted synthesis of COFs

As a proof-of-concept validation to our materials genomics methods, targeted experiments were performed to synthesize some generated COFs. Compared to the dominant activity on the synthesis of 2D-COFs, expansion of 3D-COFs remains a significantly challenging task, especially for those with topologies unreported so far. All the existing 3D-COFs were solely reported for the nets based on the GSUs with tetrahedral or octahedral geometry, leading to the materials only with six distinct 3D topologies (Table 1). In light of this, we demonstrated the targeted synthesis of two generated 3D-COFs with **ffc** topology which use the GSUs with tetragonal geometry as the core building units; such tetragonal GSUs exhibit a planar-like pattern with four connectivities, which could also be used to construct 2D-COFs^28^, in sharp contrast to those adopted for existing 3D-COFs. In addition, two 2D-COFs generated with existing **hcb** topology were also synthesized to enrich the chemistry of COFs. We noticed that among known linkages used for COFs, the most widely used one is imine bond due to its advantages such as high stability and more accessibility. More than two thirds of COFs reported in literature are fabricated through imine linkages and thus imine is more representative than other linkage models. Accordingly, imine was chosen as the linkage for the targeted materials synthesis.

### Synthesis of 3D-COFs

**Fig. 4:**
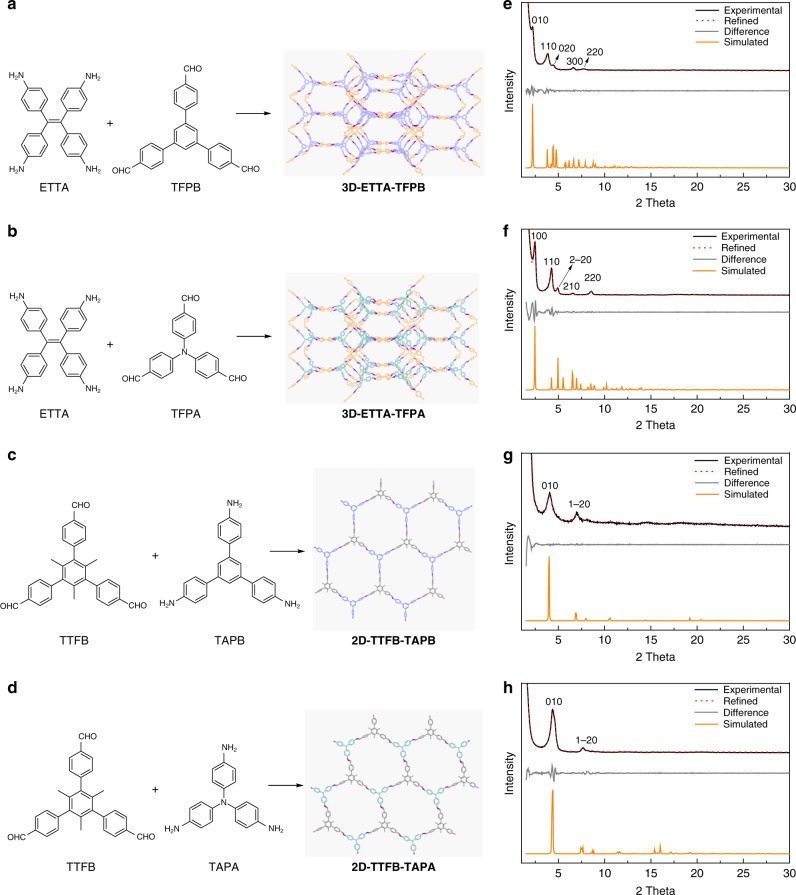
Targeted synthesis of the designed COFs. **a**–**d** The monomers used to synthesize the two 3D-COFs (**a**, **b**) and the two 2D-COFs (**c**, **d**) and their structures. **e**–**h** Comparison of the simulated and experimental PXRD patterns for the 3D-COFs (**e**, **f**) and the eclipsed 2D-COFs (**g**, **h**)

Starting from selecting monomers that can form *C*_4_ + *C*_3_ linkage, the synthesis of the two 3D-COFs with **ffc** topology, a topology which has never been experimentally achieved before, was carried out by solvothermal reactions of 4,4′,4′′,4′′′-(ethene-1,1,2,2-tetrayl)tetraaniline (ETTA) with 1,3,5-tri(4-formylphenyl)benzene (TFPB) or tris(4-formylphenyl)amine (TFPA), respectively, as shown in Fig. 4a, b. As revealed by their experimental powder X-ray diffraction (PXRD) patterns, the as-prepared 3D-COFs are highly crystalline materials. The experimentally observed PXRD pattern of **3D-ETTA-TFPB** displays two intense peaks at 2.20° and 3.84°, along with three peaks of relatively low intensities at 4.46°, 6.61° and 7.74°, which are assignable to the (010), (110), (020), (300) and (220) facets, respectively (Fig. 4e, black curve). For **3D-ETTA-TFPA**, strong PXRD peaks are observed at 2.46° (100) and 4.25° (110), accompanied by several lower peaks at 4.91° (2-20), 6.55° (210), and 8.58° (220) (Fig. 4f, black curve). In both cases, the simulated PXRD patterns (Fig. 4e, f, orange curves) of the non-interpenetrated models show good match with the experimental ones, confirming the successful synthesis of the targeted 3D-COFs with **ffc** topology. Pawley refinements (P112 space group) were performed (Fig. 4e, f, red curves), which yielded unit cell parameters of *a* = 46.1341 Å, *b* = 45.6374 Å, *c* = 20.8920 Å, *α* = *β* = 90°, γ = 120° (residuals *R*_p_ = 2.51%, *R*_wp_ = 3.53%) for **3D-ETTA-TFPB**, and *a* = 41.8782 Å, *b* = 41.2827 Å, *c* = 17.8371 Å, *α* = *β* = 90°, *γ* = 120° (residuals *R*_p_ = 4.32%, *R*_wp_ = 5.49%) for **3D-ETTA-TFPA**, nearly equivalent to those of the generated structures (Supplementary Tables 5 and 6). As indicated by the difference plots (Fig. 4e, f, gray curves), the refined PXRD patterns well reproduce the experimental data. Their FT-IR spectra showed the C=N stretching bands characteristic for imines at 1625 and 1200 cm^-1^ for the 3D-COFs, demonstrating the existence of imine units in the synthesized COFs (Supplementary Figs. 10 and 11). The solid-state ^13^C CP-MAS NMR spectra of both materials confirmed the presence of carbon atoms of the imine linkages by the appearance of the peaks around 156 ppm (Supplementary Figs. 12 and 13).

### Synthesis of 2D-COFs

To further verify the proposed genomic strategy for COF construction, we also prepared two 2D-COFs with **hcb** topology, which were generated using the GSUs both with *C*_3_-symmetric configuration. One material was synthesized by the reaction of 1,3,5-trimethyl-2,4,6-tris(4-formylphenyl)benzene (TTFB) and tris(4-aminophenyl)benzene (TAPB), while the other one was obtained by the condensation of TTFB and 1,3,5-tris(4-aminophenyl)amine (TAPA) (Fig. 4c, d). Similar to the above 3D-COFs, the targeted 2D-COFs were also produced as crystallites under solvothermal conditions. The experimental PXRD patterns of the as-prepared materials were collected and compared with the simulated ones, which confirmed the expected crystal structures of **2D-TTFB-TAPB** and **2D-TTFB-TAPA** in eclipsed stacking mode, as indicated by the good agreements between the experimental PXRD and simulated data (Fig. 4g, h). Refined PXRD patterns from Pawley refinements match the experimental data well (Fig. 4g, h, gray curves), which gave lattice parameters of *a* = 25.9255 Å, *b* = 25.3467 Å, *c* = 4.8289 Å, *α* = *β* = 90°, *γ* = 120° (residuals *R*_p_ = 4.54%, *R*_wp_ = 5.54%) for **2D-TTFB-TAPB**, and *a* = 24.4371 Å, *b* *=* 23.1107 Å, *c* = 5.6147 Å, *α* = *β* = 90°, *γ* = 120° (residuals *R*_p_ = 3.50%, *R*_wp_ = 4.72%) for **2D-TTFB-TAPA** (Supplementary Tables 7 and 8). The FT-IR and ^13^C NMR results also support successful synthesis of the COFs (Supplementary Figs. 14–19).

The above proof-of-concept experiments reveal the reliability of the materials genomics-based algorithms proposed in this work as well as its powerfulness. In contrast to an easy understanding of the topology of 2D-COFs by manually plotting the structures on the paper, it is hard to intuitionally figure out what architecture will be formed for 3D-COFs from different GSUs, especially in the finding of materials with unreported topologies. The above successful synthesis of 3D-COFs demonstrates the feasibility of our computational strategy in guiding experimental efforts to develop nanoporous materials.

**Fig. 5:**
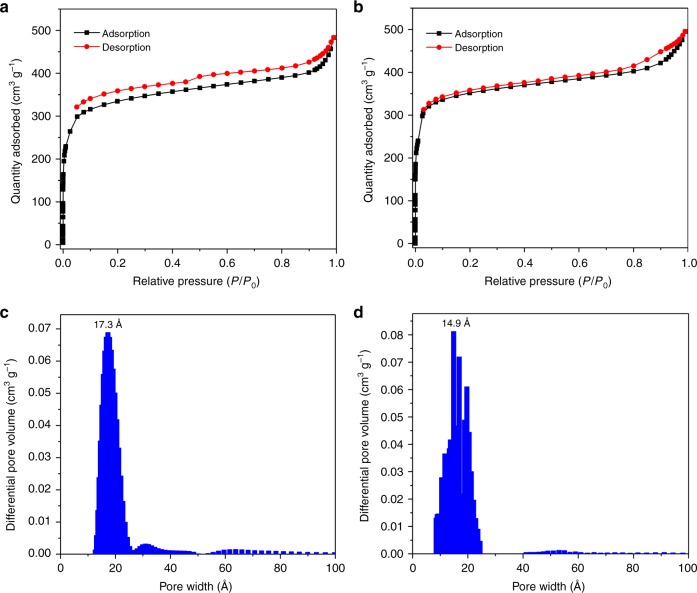
Gas adsorption. N_2_ adsorption isotherms (77 K) of **a** **3D-ETTA-TFPB** and **b** **3D-ETTA-TFPA**. Pore size distribution profiles of **c** **3D-ETTA-TFPB** and **d** **3D-ETTA-TFPA**

Nitrogen adsorption-desorption measurements were further carried out to assess the as-prepared COFs (Fig. 5a, b, and Supplementary Fig. 20a, b). Their BET surface areas were calculated based on the N_2_ adsorption data in the range of *P/P*_0_ between 0.05 and 0.2 (Supplementary Fig. 21). For the 3D-COFs, surface areas of 1174 and 1202 m^2^ g^-1^ were estimated for **3D-ETTA-TFPB** and **3D-ETTA-TFPA**, respectively, with total pore volumes (at *P*/*P*_0_ = 0.99) being 0.75 cm^3^ g^−1^ for the former and 0.77 cm^3^ g^−1^ for the latter. Their pore size distributions (PSDs) were estimated through nonlocal density functional theory (NLDFT), which display main distributions around 1.73 and 1.49 nm for **3D-ETTA-TFPB** and **3D-ETTA-TFPA**, respectively, indicating a good degree of crystallinity (Fig. 5c, d). In the case of the 2D-COFs, the same procedures afforded BET surface areas, pore volume (at *P*/*P*_0_ = 0.99) and pore size to be 1526 and 2448 m^2^ g^-1^, 1.38 and 1.56 cm^3^ g^−1^, and 2.05 and 1.48 nm for **2D-TTFB-TAPB** and **2D-TTFB-TAPA**, respectively (Supplementary Fig. 20c, d). The observed pore sizes well match the theoretically predicted ones.

## Discussion

In this work, a concept of the GSUs with reactive sites was proposed for the gene partition of COFs and a construction method of the QReaxAA was developed for structure generation by mimicking COFs natural growth processes. The genomic COF construction methods based on them can efficiently generate COFs to meet the requirement of high-throughput computational materials design, and the generation of structures with unreported topologies would facilitate greatly experimental endeavors on their synthesis. The targeted synthesis of two 3D-COFs and two 2D-COFs highlights the usefulness and the reliability of the methods. As a result, this work not only provides useful methods and tools for high-throughput materials construction, but also will contribute to the switch of materials development mode, making the materials development greener and more efficient.

## Methods

### Position method for the GSUs

To conveniently generate a variety of COFs, we adopted three different geometrical positioning methods for the connection of GSUs, as shown in Supplementary Fig. 2. Three non-colinear positioning atoms are derived for each reactive site on the GSUs. For structure construction, the choice of positioning method depends on the specific center-type GSUs. The first method (Supplementary Fig. 2a) is applicable to the situation that the three positioning atoms at each reactive site of the GSUs are self-determined. It means that the so-obtained structures have distinct boundaries between center and linker GSUs, just analogous to those between inorganic and organic building blocks defined for MOFs. The second method is used to deal with the center GSUs that two positioning pseudo-atoms at each reactive site need to be consulted with linker GSUs; i.e., it is linker-dependent. This method can be employed to design structures analogous to ZIF-8 with a sodalite topology^29^ and so on (Supplementary Fig. 2b). The last one is proposed to assemble structures with infinitely-extending chains that consist of the same center GSUs, for example, those analogous to the inorganic chains in the MOFs like MIL-47^9^.

### Linkage principle between the GSUs

As described above, three positioning methods were used for the connection of the GSUs. No matter what kind of positioning method is used, our QReaxAA algorithm will eventually convert geometrical information of the three positioning atoms located at each reactive site into the unit vectors defined by three virtual points using the green stars shown in Supplementary Fig. 3, allowing to assemble COF structures conveniently using mathematical operations. To briefly demonstrate the linkage principle between the GSUs, we take the connection between the reactive site 1 of one center GSU (Supplementary Fig. 3a) and the reactive site 4 of one linker GSU (Supplementary Fig. 3b) as an example. Initially, the center GSU is placed anywhere in the space. The linker GSU is then added and its position is adjusted using a rotation matrix and a translation vector so that the reactive site 4 is oriented correctly to the reactive site 1. When the coordinates of stars 4 and 5 are respectively coincident with those of stars 1 and 2 and the vector 4→6 is in parallel with the vector 1→3, the two reactive sites are considered to connect successfully. The linking processes for other reactive sites as well as other GSUs are essentially the same. When all the reactive sites are linked and the periodic boundary conditions are found, overall framework of the crystal structure is generated.

### Procedure of structure generation

Supplementary Fig. 4 shows the general flowchart implemented in our method for enumerative generation of COFs. At the beginning, one kind of center GSUs and at most two kinds of linker GSUs are selected each time, which are stepwise combined together. If an atomic overlap occurs during the connection, our algorithm will go backwards and retry the rest of reactive sites or other kinds of GSUs. If repetitive connectivity emerges in one direction, a periodic boundary will be imposed along this direction to connect the GSUs, instead of adding more GSUs. For 3D-COFs, three periodic boundaries need to be found. For 2D-COFs, while two periodicities can be found from the connection of GSUs, an arrangement of interlayer spacing is required prior to imposing the third one. After successful generation of parent structures, chemical modification will be performed by attempting all kinds of functional groups. To accelerate the assembling process, some constraints are additionally specified in our algorithms to avoid very long time on attempting infeasible combinations of GSUs. Specifically, with a given selection of GSUs, if attempting to connect GSUs exceeds 5000 steps or the number of reactive sites need to be linked is larger than 300, current selection will be discarded and other GSUs will be chosen from the library to conduct next generation. We also perceived that the at least 95% of 2D-COF structures experimentally reported so far dominantly adopt eclipsed stacking mode, and interpenetration behaviors in 3D-COFs can be affected by many factors such as synthesis conditions^30,31^. Thus, the COF database presented in this work only contains the unique structures featuring no mutually interdigitated frameworks.

### Self-adaption algorithm for interlayer-spacing determination

The covalently-bonded framework of 2D-COFs is restricted to 2D sheets, which are stacked together via van der Waals forces to form a laminar structure. Thus, there is no connection between the GSUs in adjacent layers. By examining the structures of synthesized 2D-COFs, we found that the interlayer spacing of most materials essentially is in the range of 3.0–4.0 Å. However, for some materials like IL-COF-1^32^ and TD-COF-5^33^, the interlayer spacing can reach 6.6 and 7.5 Å respectively, which is due to the fact that their structures contain non-planar sheets. Thus, it is impossible to unitarily use a fixed mean value for high-throughput generation of 2D-COF structures. To address this issue, a self-adaption algorithm was adopted in our genomics-based method, in which the interlayer spacing (*d*) was set equal to the largest contribution between center and linker GSUs plus that of functional group, as given by

1 $$d = \max \left( {d_{{\mathrm{center}}},d_{{\mathrm{linker}}}} \right) + d_{{\rm{functional}}\,{\rm{group}}},$$

where *d*_center_, *d*_linker_ and *d*_functional group_ are the interlayer-spacing contributions of the center, the linker, and the functional-group GSUs that constitute the targeted structure, respectively.

The contributions of existing center and linker GSUs were determined from the interlayer-spacing information of the synthesized COFs. For other center and linker GSUs, their contributions were inferred from these derived GSUs with similar configurations. Since the functionalized COFs reported experimentally are very scarce, it is hard to establish the contributions of functional groups to interlayer spacing. With respect to this issue, we approximately set the contributions of them equal to the differences between the interlayer spacing of functionalized and parent COFs on the basis of the structures optimized using molecular mechanics. For this purpose, five 2D-COFs reported experimentally (COF-5^34^, COF-10^35^, COF-LZU1^36^, CTF-1^37^ and CTF-2^38^) were selected and each of them was modified using the eight kinds of functional groups considered in this work. All the built structures were optimized using the Forcite module of Materials Studio. The final contribution of each functional group was averaged from its contributions to these five COFs, as listed in Supplementary Table 1. To gain an intuitive understanding on the self-adaption algorithm is realized in our method, we provided an example in Supplementary Fig. 5 to outlines the steps for the construction of a 2D-COF and its functionalized form. To computationally determine the specific stacking mode between the layers of 2D-COFs, it is necessary to conduct higher level of theoretical methods such as quantum mechanics calculations, which however are very time consuming to be applied to the large number of 2D structures in our database. Also, most of the 2D-COFs reported so far are presented with eclipsed structures, and the eclipsed and staggered modes do not have large difference in interlayer space. At the same time, both the stacking modes have no influence on the connections between monomers and the resulting topologies. Thus, as a primary step, contributions of various GSUs to interlayer spacing were determined on simplistic model of perfect eclipsed stacking structures, which were further adopted to perform high-throughput construction of 2D-COFs. Since there are some experimental 2D-COF structures with layers exhibiting slightly offset arrangements, in the future we will consider such effects during the improvement of our genomics method so as to make more reasonable 2D-COF structures.

### Molecular mechanics optimizations

To validate proposed computational protocol, we performed the geometry optimizations for some typical COFs using the Smart algorithm implemented in the Forcite module of Materials Studio software. This algorithm is a cascade of the steepest descent, adjusted basis set Newton-Raphson, and quasi-Newton methods. The DREIDING force field^39^ combined with the QEq charge equilibration method^40^ were used to describe the bonded and non-bonded interactions between atoms during the optimization processes. Both cell parameters and atomic positions were allowed to fully relax until the total energies of the structures were minimized.

### Geometric analysis of COF structures

Structural features of the generated COFs in our database were analysed using the Zeo + + code^41^. It is a powerful tool for performing high-throughput geometry-based analysis of porous materials, including the calculations of largest-cavity diameter (LCD), pore-limiting diameter (PLD), accessible surface area (*S*_acc_) and free volume (*V*_free_). In this work, accessible surface area of each material was calculated by a probe molecule with a size equal to the kinetic diameter of N_2_ (3.68 Å), while a probe size of 0.0 Å was applied to calculate the free volume which is the absolute amount of volume not occupied by the framework atoms. The void fraction (*ϕ*) was determined from the ratio of free volume to the total volume of the cell. The program used for the topological analysis is the open source tool TOPOS^42^.

### Validation of the generated COF structures

To validate our genomic approach for the construction of COF structures, we compared some intrinsic structural features of 10 synthesized COFs (6 2D-COFs and 4 3D-COFs) with those of the built structures and the built structures after molecular mechanics optimization. Supplementary Table 2 shows a comparison of the cell parameters of these materials, together with Supplementary Table 3 for their crystal densities (*ρ*_crys_), pore-limiting-diameters (PLD), largest-cavity diameters (LCD), accessible surface areas (*S*_acc_) and void fractions (*ϕ*). It can be found that these structural descriptors are in good agreement for each examined material. In addition, to validate the applicability of the interlayer-spacing contributions of functional groups used in our self-adaption algorithm, we arbitrarily selected 8 functionalized 2D-COFs that are not included in the training set and examined the variation of the cell parameters of each built structure after molecular mechanics optimization. As evidenced from the results shown in Supplementary Table 4, the cell length along *c* direction for each material changes only marginally, demonstrating that the contributions of the functional groups given in Supplementary Table 1 are suitable to determine the interlayer spacing of functionalized 2D-COFs.

Our method was mainly based on the monomers and the reaction types adopted in the synthesis of existing COFs, and thus all the structures were achieved through plausibly linking diverse monomers. In regard to examining whether certain COF structures that are likely to form, higher levels of theoretical approaches (such as quantum mechanics calculations) were usually used in literature to explore their thermodynamic feasibility. However, not only is there a great difficulty in applying them to hundreds of thousands of COF structures in our database, but whether one material can ultimately be formed is also affected by kinetic factors relevant to solvents and synthesis conditions, which are difficult to consider in calculations.^26^ Consequently, similar to the large databases computationally reported for MOFs and other materials, it cannot be guaranteed that all the constructed COF structures can be realized experimentally. However, apart from the offer of a theoretical guidance for the synthesis of COFs, the database also can provide a useful foundation for studying the structure-property relationships of COFs towards interested applications. Considering the issues described above, in the future we will manage to improve our algorithms to provide some useful insights into the synthetic conditions required to make COFs.

### Synthesis of **3D-ETTA-TFPB**

This COF was prepared by adding 4,4′,4′′,4′′′-(ethene-1,1,2,2-tetrayl)tetraaniline (ETTA) (35.4 mg, 0.09 mmol), 1,3,5-tris(4-formylphenyl)benzene (TFPB) (46.8 mg, 0.12 mmol), chlorobenzene (3 mL) and acetic acid (aq., 9 M) (0.3 mL) into a glass ampoule. After being degassed through three freeze-pump-thaw cycles and then flame-sealed under vacuum, the ampoule was heated at 120 °C in an oven for three days. The resulting precipitate was collected through filtration, washed three times with anhydrous THF and 1, 4-dioxane, and immersed in anhydrous THF (10 mL) for 1 h during which the solvent was decanted and replenished with fresh THF three times. The product was filtrated and then dried under dynamic vacuum at 120 °C for 2 h to afford a yellow powder (60.8 mg, 80%).

### Synthesis of **3D-ETTA-TFPA**

This COF was prepared as an orange powder (40 mg, 88%) using the same synthetic protocol of **3D-ETTA-TFPB** from ETTA (23.6 mg, 0.06 mmol) and tris(4-formylphenyl)amine (TFPA) (26.4 mg, 0.08 mmol) in a mixture of chlorobenzene (2 mL) and acetic acid (aq., 9 M) (0.2 mL).

### Synthesis of **2D-TTFB-TAPB**

This COF was prepared as a light yellow powder (50 mg, 76%) using the same synthetic protocol of **3D-ETTA-TFPB** from 1,3,5-trimethyl-2,4,6-tris(4-formylphenyl)benzene (TTFB) (39 mg, 0.09 mmol) and tris(4-aminophenyl)benzene (TAPB) (31.5 mg, 0.09 mmol) in a mixture of chlorobenzene (3 mL) and acetic acid (aq., 9 M) (0.3 mL).

### Synthesis of **2D-TTFB-TAPA**

This COF was prepared as a gold powder (27 mg, 67%) using the same synthetic protocol of **3D-ETTA-TFPB** from TTFB (26 mg, 0.06 mmol) and 1,3,5-tris(4-aminophenyl)amine (TAPA) (17.4 mg, 0.06 mmol) in a mixture of 1,2-dichlorobenzene (2 mL) and acetic acid (aq., 9 M) (0.2 mL).

### Characterizations of COFs

Powder X-ray diffraction measurements were carried out with an X’Pert PROX system using monochromated Cu/Kα (*λ* = 0.1542 nm). Each sample was spread on the square recess of XRD sample holder as a thin layer. Solution-state ^1^H- and ^13^C NMR spectra were collected on a Bruker Fourier 500 M or a 400 M spectrometer. Solid-state ^13^C cross-polarization/magic angle spinning (CP/MAS) spectra were collected on an Agilent DD2 600 Solid system equipped with a 3.2 mm HFXY MAS probe. The Hartmann-Hahn conditions of the CP experiment were obtained at a15 kHz MAS spinning speed with a contact time of 2.0 ms. Recycle delay times are 5 s. Fourier transform infrared spectroscopy (FT-IR) were obtained by using a Nicolet iS10 spectrometer.

### Nitrogen adsorption-desorption isotherm measurements

The measurements were carried out using a Quantachrome Autosorb-IQ instrument. Before gas adsorption measurements, the as-prepared samples (∼50 mg) were activated by being immersed in anhydrous dioxane for 12 h. The solvent was decanted and the samples were dried under dynamic vacuum at 160 °C for 8 h. The resulting samples were then used for gas adsorption measurements from 0 to 1 atm at 77 K. The Brunauer-Emmett-Teller (BET) method was utilized to calculate the specific surface areas. By using the non-local density functional theory model, the pore size distributions were derived from the adsorption data.

### Code availability

The code used to generate the COF structures are available from the corresponding authors upon reasonable request.

## Electronic supplementary material

Supplementary Information

## Data Availability

All relevant data that that support the findings of this study are available from the corresponding authors upon request.
