# Supplementary material for: Materials genomics methods for high-throughput construction of COFs and targeted synthesis
Source: Nat Commun. 2018 Dec 10;9:5274. doi: 10.1038/s41467-018-07720-x (PMC6288119; doi:10.1038/s41467-018-07720-x)
Supplement: Supplementary file 1 — Supplementary Information [file 41467_2018_7720_MOESM1_ESM.pdf]

Supplementary Information

Materials genomics methods for high-throughput construction of COFs and targeted synthesis

Lan et al.

Supplementary Note 1

Library of the genetic structural units. From the knowledge of common reaction types used for COF synthesis[1-4], a concept of genetic structural units (GSUs) with reactive sites was proposed in this work to partition the genes of COFs, leading to a library of 130 GSUs with a variety of geometries, number of reactive sites and chemical compositions. These GSUs are composed of three types: center, linker and functional group, as listed in Supplementary Figure 1. While most of the GSUs are derived from the existing COF materials, some center-type GSUs are designed to generate COFs with new topological networks, together with some linker- and functionalization-type GSUs adopted from experimentally reported MOFs.

a

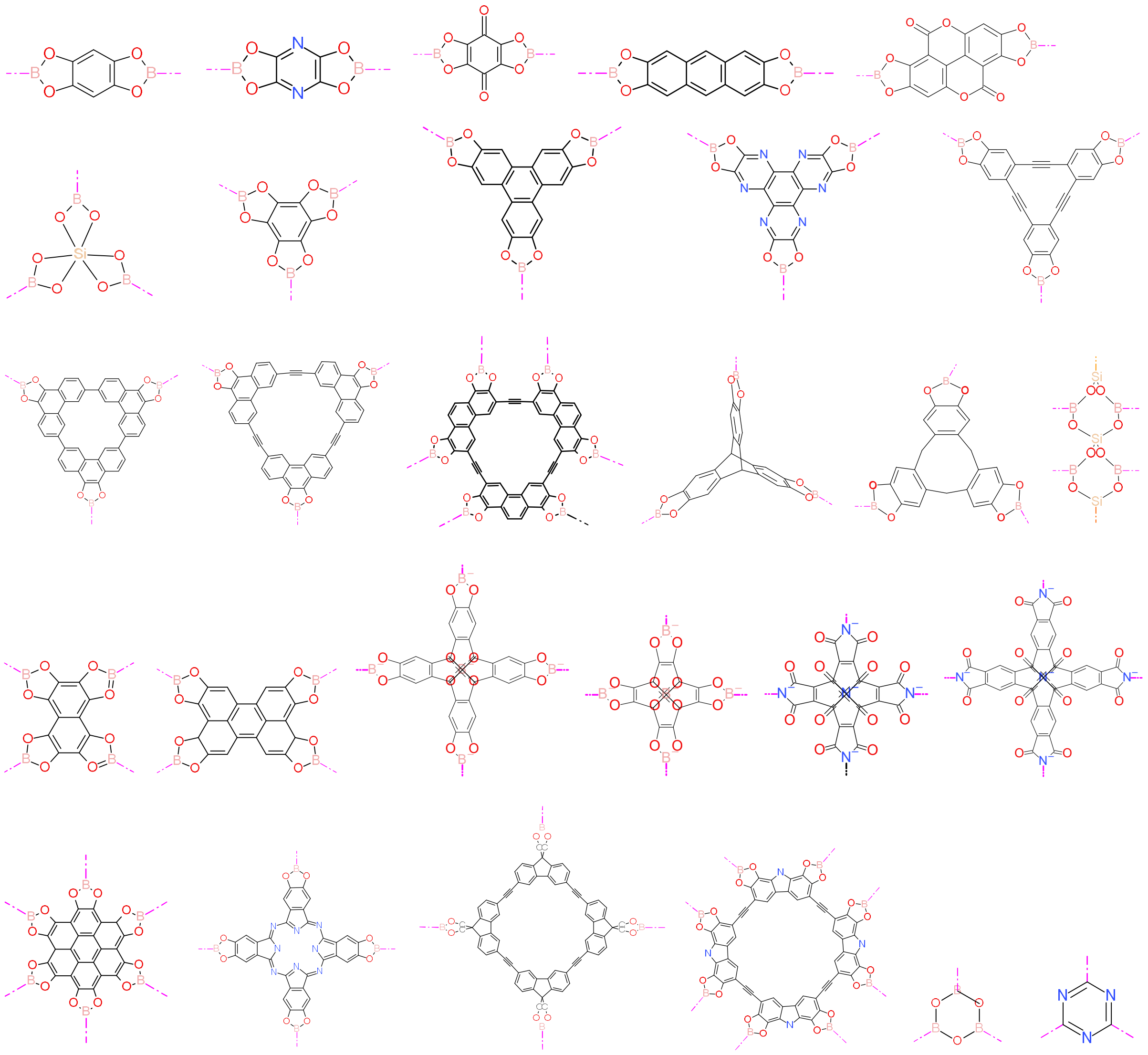

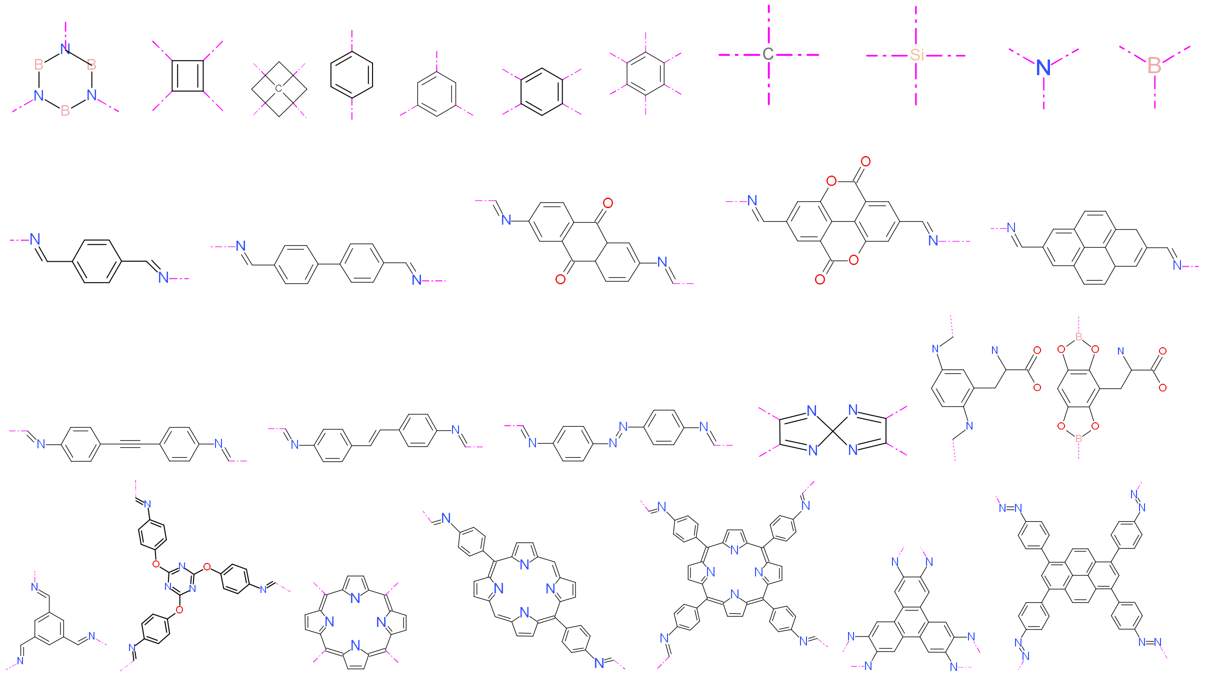

b

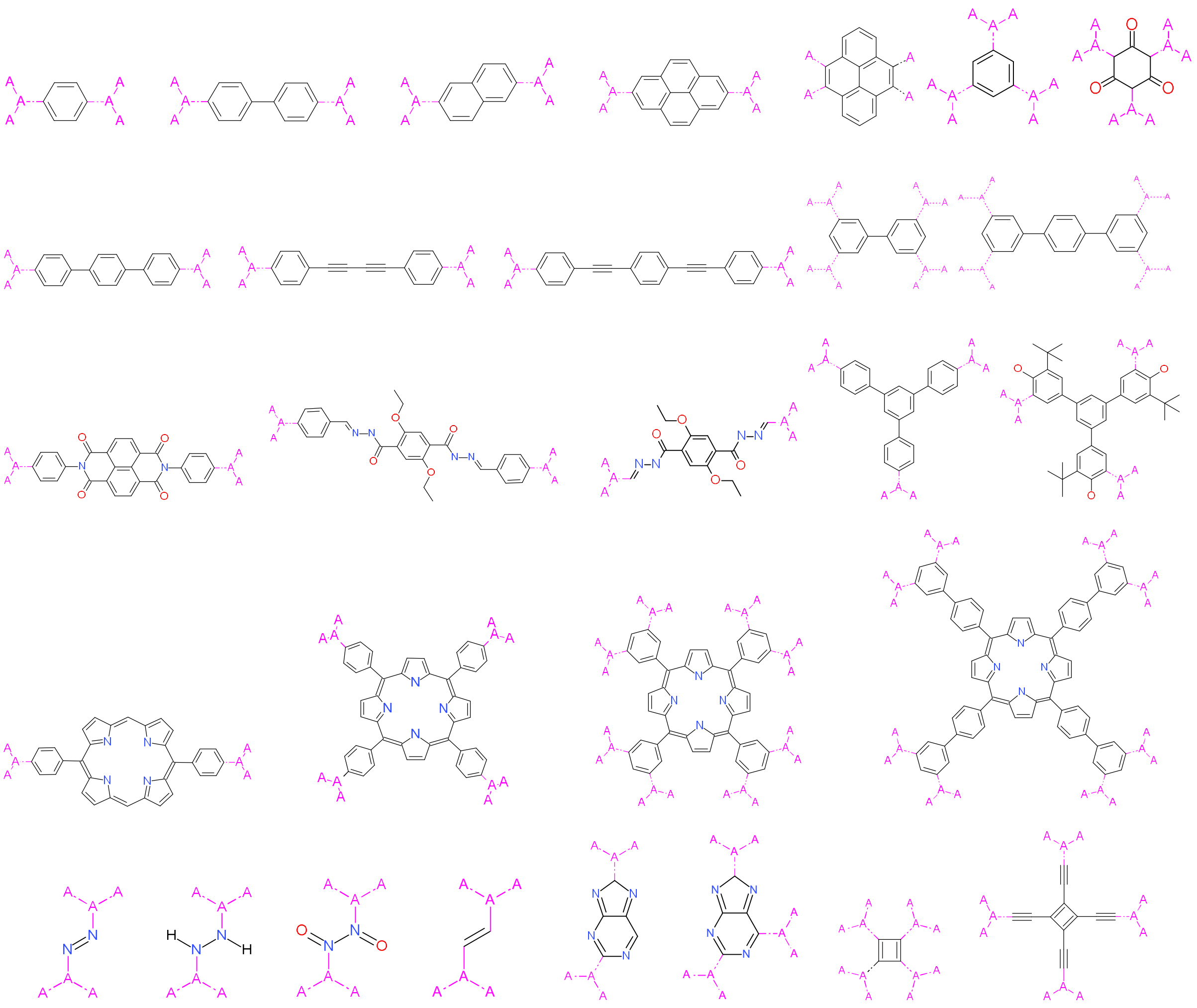

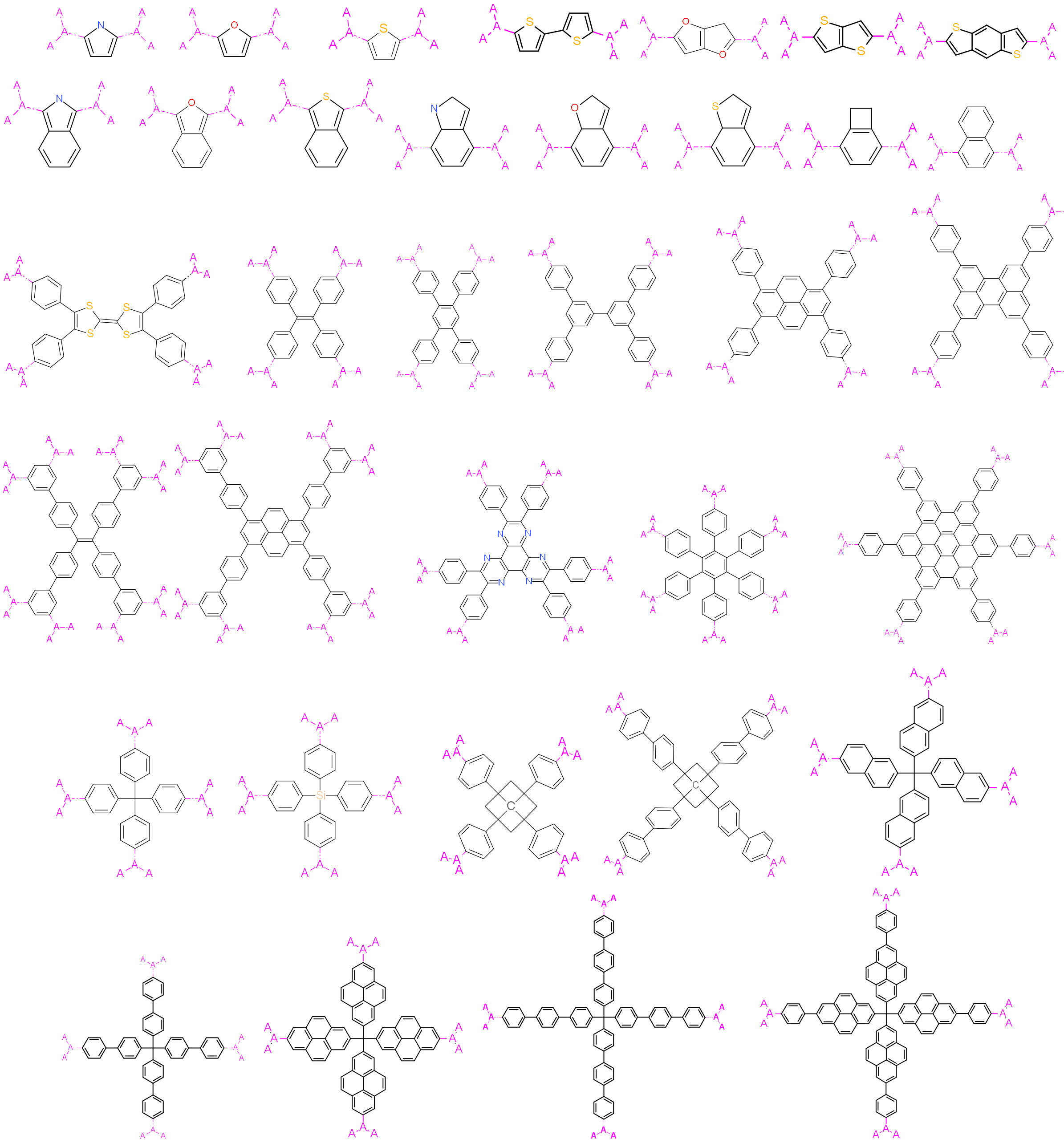

c

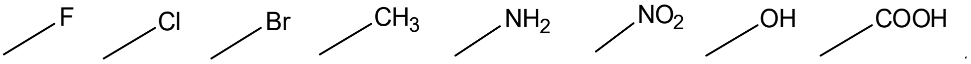

Supplementary Figure 1 | Full list of the GSUs used to generate the database of COFs. a, 58 centers. **b**, 64 linkers. **c**, 8 functional groups. The pink dashed lines in the center-type GSUs and the pink "A" characters in the linker-type GSUs represent the information of the predefined reactive sites, and they will all be eliminated after successful connection. According to the chemical reaction types reported for synthesis of COF-type materials, the "AAA" notations can stand for BOO, CNN, CNH, NBB and CCC.

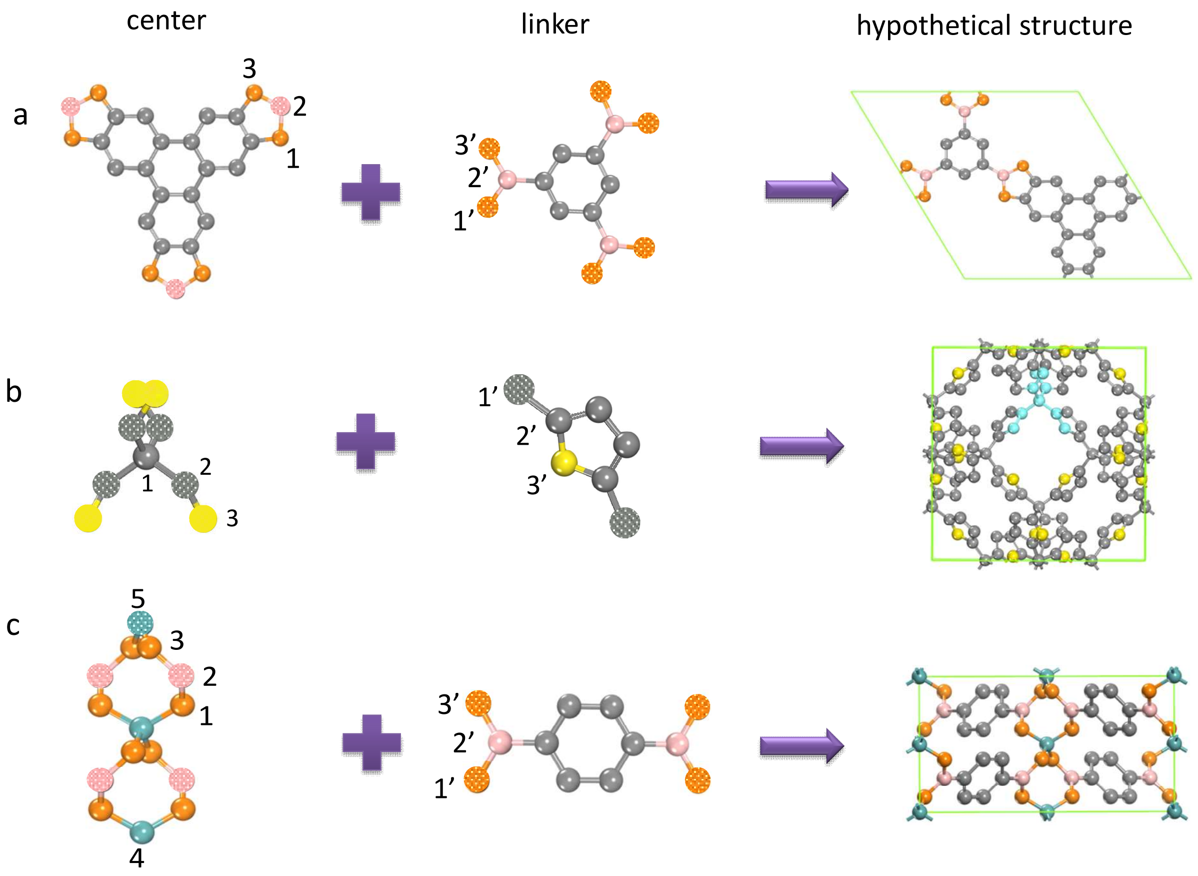

Supplementary Figure 2 | Three different positioning methods for the connection of GSUs. The positioning atoms marked by 1, 2 and 3 in center GSUs respectively correspond to the positioning atoms 1’, 2’ and 3’ in linker GSUs. After successful positioning, the solid atoms will be reserved while the textural atoms will be removed. **a**, The three positioning atoms at each reactive site of the center GSUs are self-determined, and the textural atoms of linker GSUs are just used for positioning. **b**, Only one positioning atom at each reactive site of the center GSUs is solid and the other textural two are dependent on the linker GSUs to be connected. **c**, The positioning method for atoms 1, 2, and 3 is similar to the first one, but the atoms 4 and 5 are self-periodically connected. Grey, orange, yellow, olive and pink spheres represent C, O, S, Si and B atoms, respectively. H atoms are omitted for clarity.

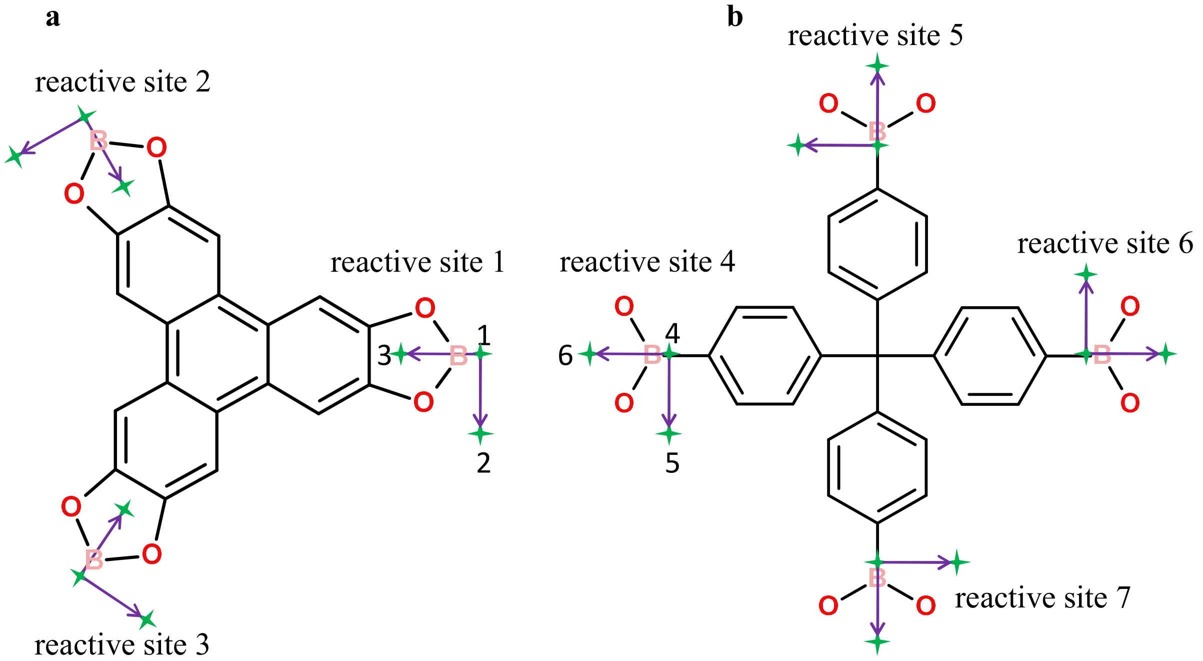

Supplementary Figure 3 | Definition of the reactive sites by virtual points. a, One kind of center GSUs with three reactive sites. **b**, One kind of linker GSUs with four reactive sites. The green stars are the positioning virtual points and the purple arrows are the positioning unit vectors.

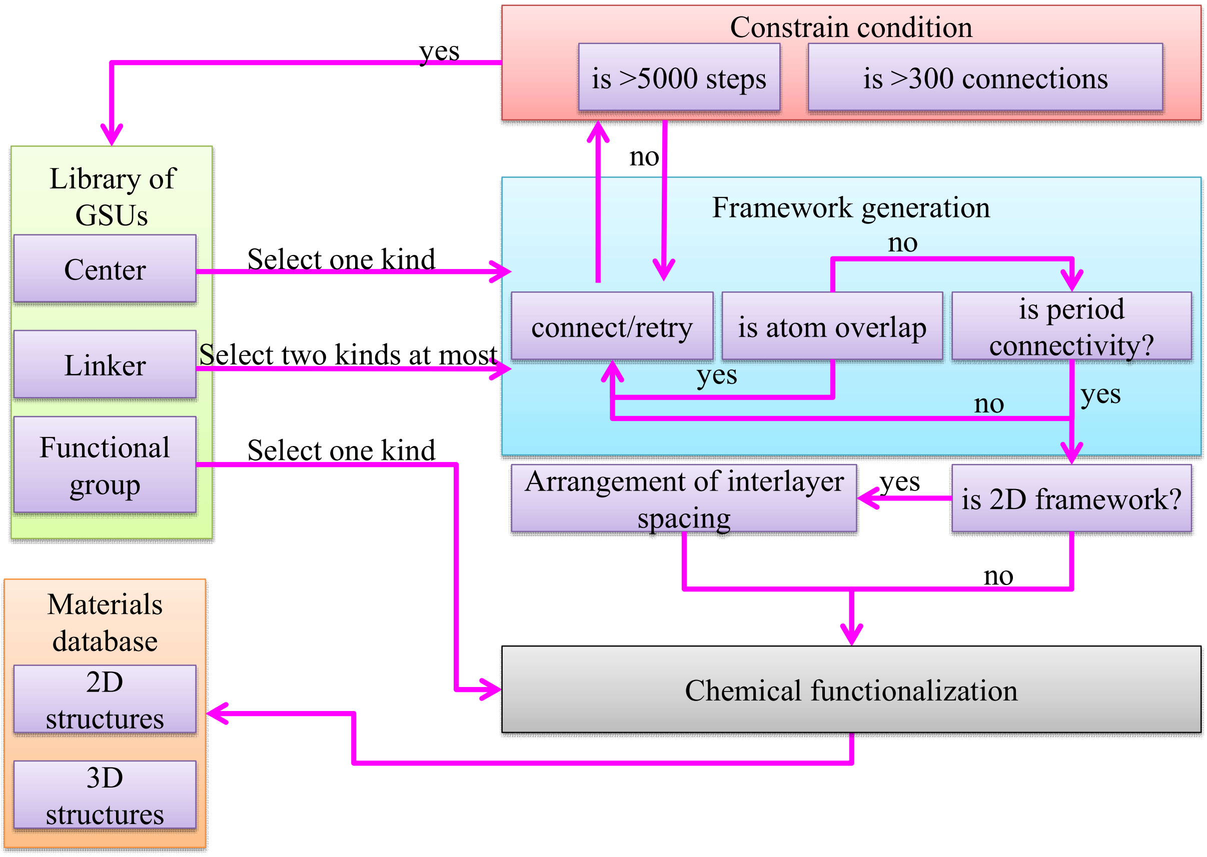

Supplementary Figure 4 | A general flowchart implemented in our genomics-based QReaxAA method for enumerative generation of 2D- and 3D-COFs from a library of the GSUs.

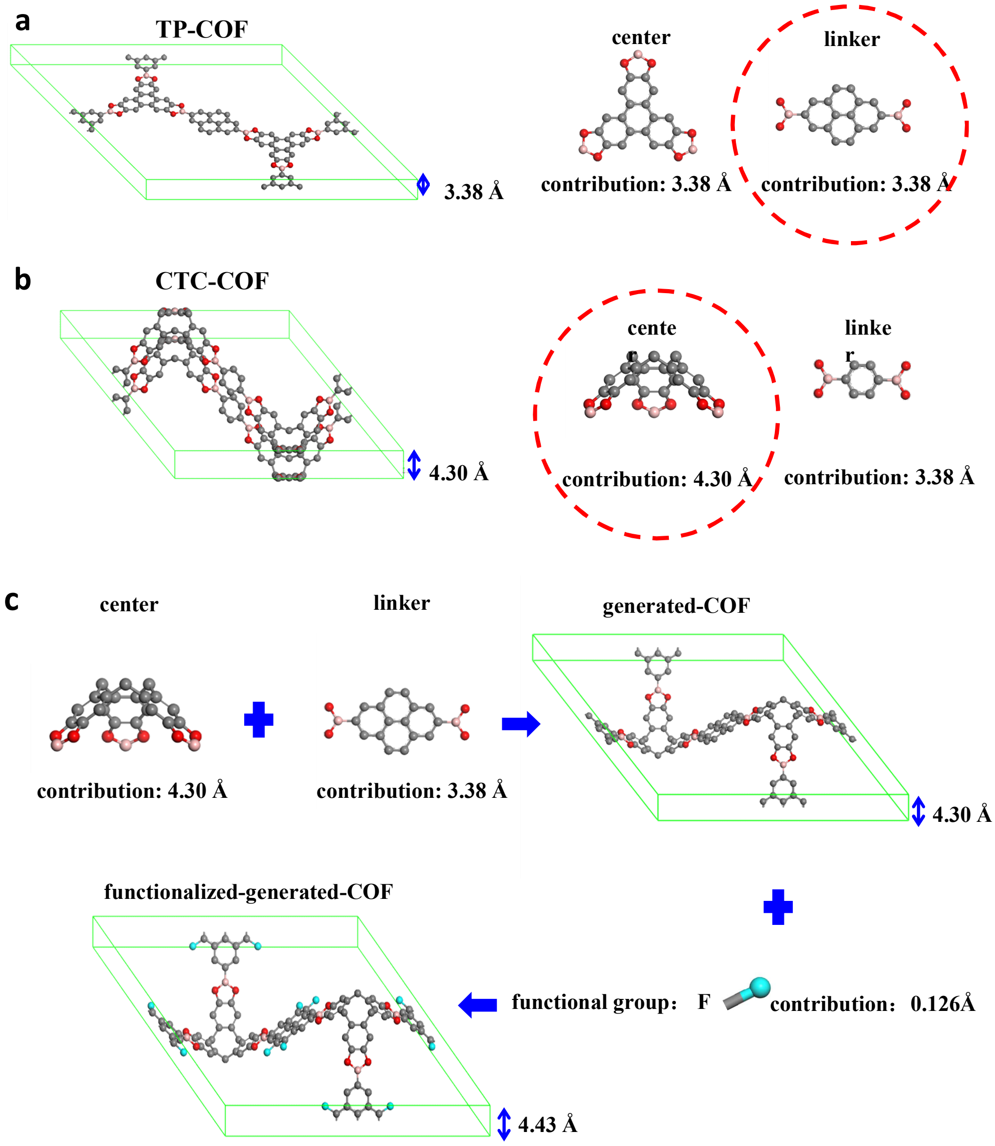

Supplementary Figure 5 | Illustration of the principle of the self-adaption-algorithm for constructing 2D-COFs. a, The interlayer spacing of TP-COF[5] reported experimentally is 3.38Å. Since the center and linker GSUs are planar, both the contributions of them are set to 3.38 Å. **b**, The linker GSU of the synthesized CTC-COF[6] has a planar configuration similar to that of TP-COF, thus its contribution can be set to 3.38 Å. Since the center GSU is non-planar and dominates the interlayer spacing, its contribution is equal to the interlayer spacing (4.30 Å) of this material. **c**, Assembly of a new 2D-COF from the linker GSU of TP-COF and the center GSU of CTC-COF. The interlayer spacing of the generated-COF is equal to the larger contribution of the center GSU (4.30 Å). For its F-functionalized form, the interlayer spacing is approximately set to 4.43 Å by adding the contribution of this functional group (0.126 Å).

Supplementary Table 1. Contribution of functional-group GSUs to the interlayer spacing of 2D-COFs.

**Functional group**	$d_{\text{functional group}}$ **(Å)**
–F	0.126
–Cl	0.186
–Br	0.138
$–CH_3$	0.478
$–NH_2$	0.092
–OH	0.096
–COOH	0.223
$–NO_2$	0.296

2D

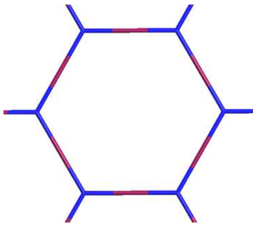

(3,2), **hcb**
hexagonal

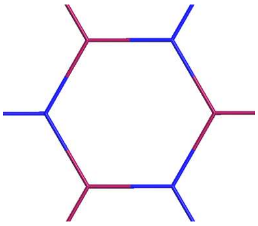

(3,3), **hcb**
hexagonal

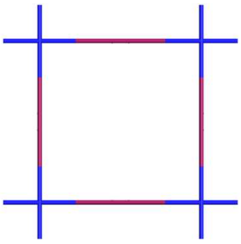

(4,2), **sql**
tetragonal

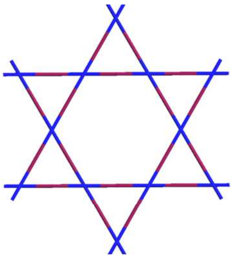

(4,2), **kgm**
hexagonal/triangular

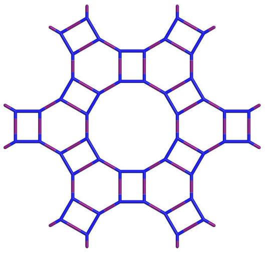

(4,2), **fxt**
dodecagonal/hexagonal
/tetragonal

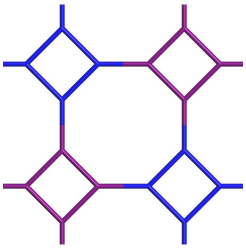

(4,4), **fes**
octagonal/tetragonal

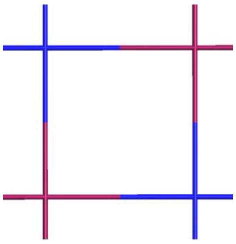

(4,4), **sql**
tetragonal

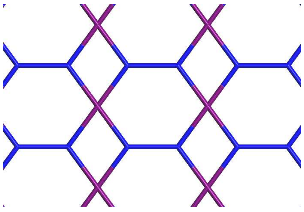

(4,4), **bex**
hexagonal/tetragonal

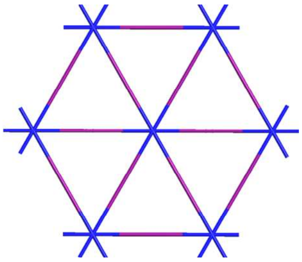

(6,2), **hxl**
triangular

3D

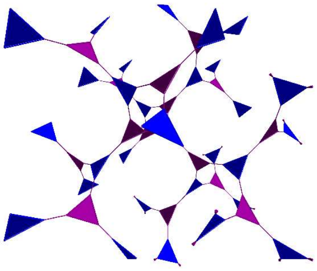

(3,3), **srs**

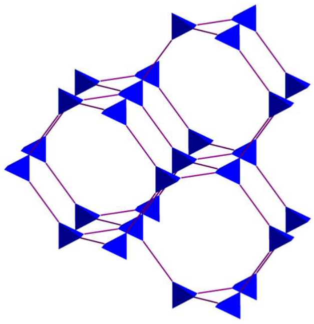

(4,2), **dia**

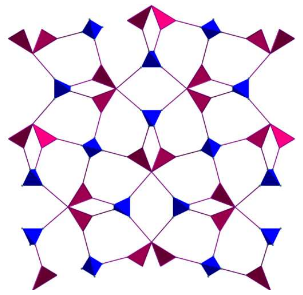

(4,3), **ctn**

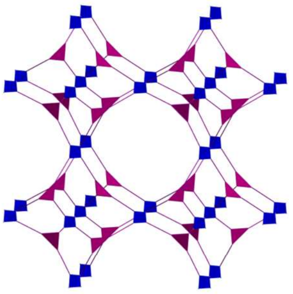

(4,3), **bor**

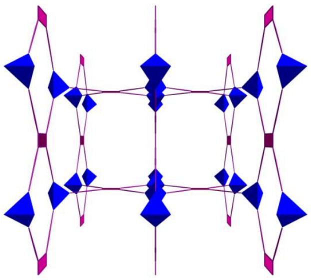

(4,4), **pts**

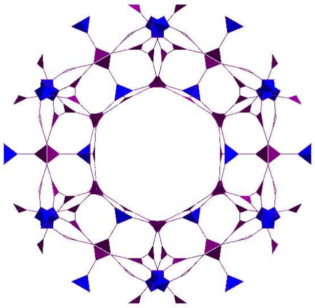

(12,4), **rra**

Supplementary Figure 6 | Linkages and topologies of existing 2D- and 3D-COFs.

2D

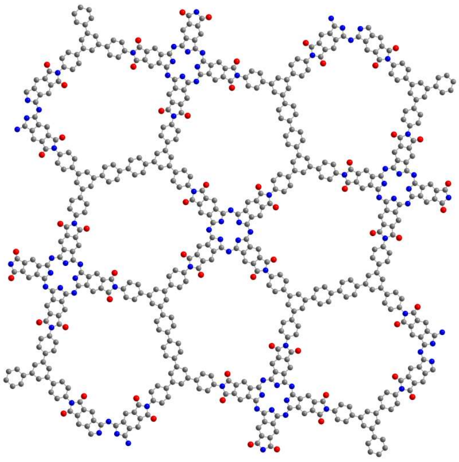

(4,4), **mcm**, GCOF-BP

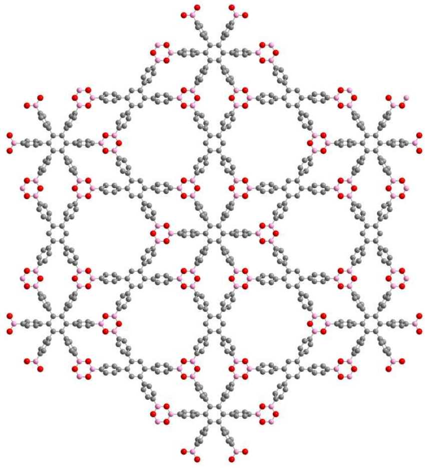

(6,4), **tth**, GCOF-HT

3D

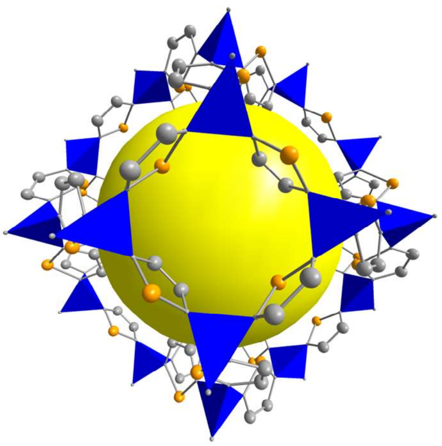

(4,2), **sod**, GCOF-MT

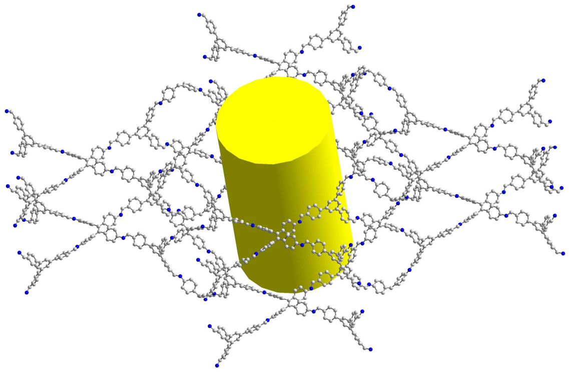

(4,3), **ffc**, 3D COF-ETTA-TBDB

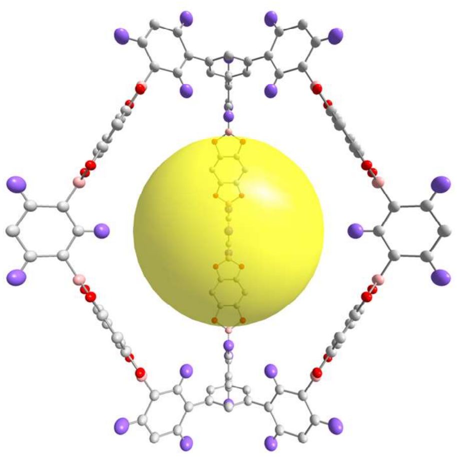

(6,2), **acs**, GCOF-TT

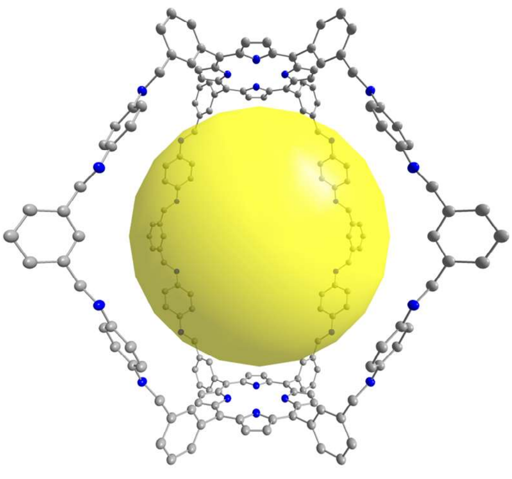

(8,2), **bcu**, GCOF-OD

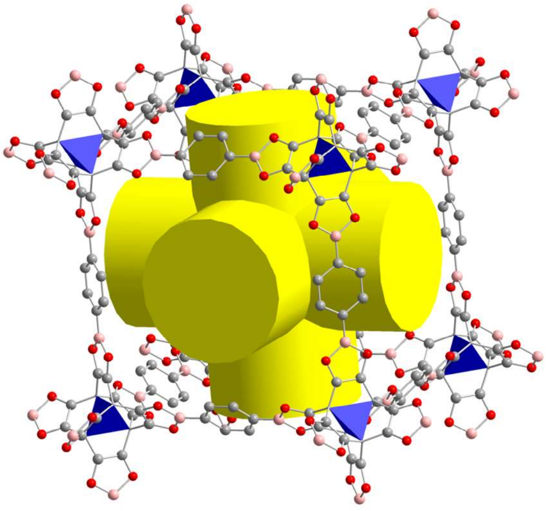

(6,2), **pcu**, GCOF-HB

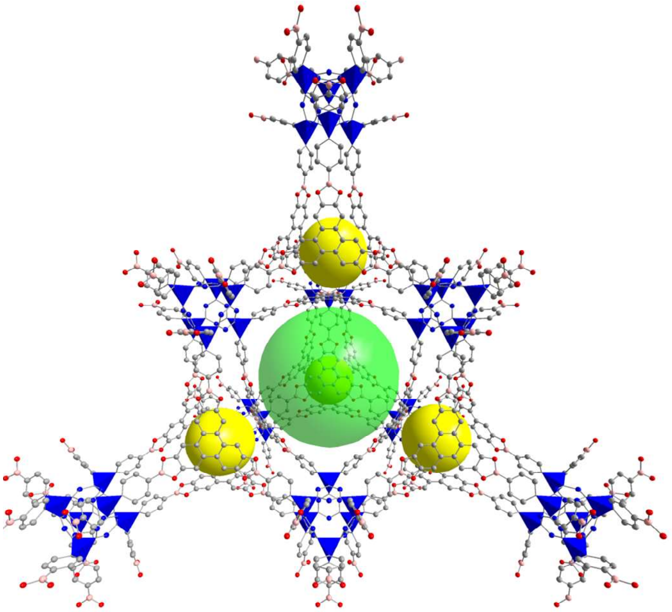

(12,3), **ttt**, GCOF-DH

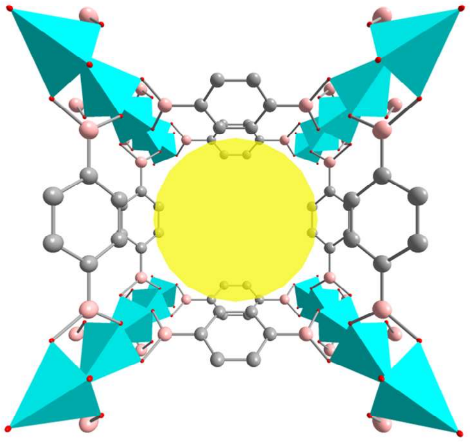

(4,2), **cds**, GCOF-SB

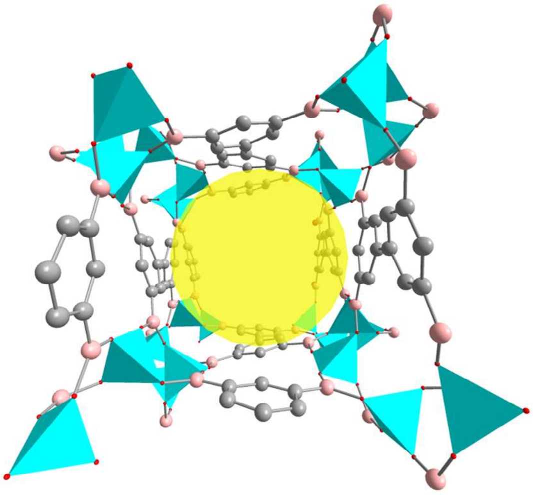

(4,4), **cda**, GCOF-ST

Supplementary Figure 7 | Some respresentatives of the generated 2D- and 3D-COFs with new topologies. Under each structure, there is a label composed of linkage, topology symbol and structure name. Grey, red, blue, orange, pink and purple spheres represent C, O, N, S, B atoms and methyl groups, respectively. The blue and cyan polyhedrons represent the C-C and Si-O tetrahedrons, respectivily. The H atoms are omitted for clarity.

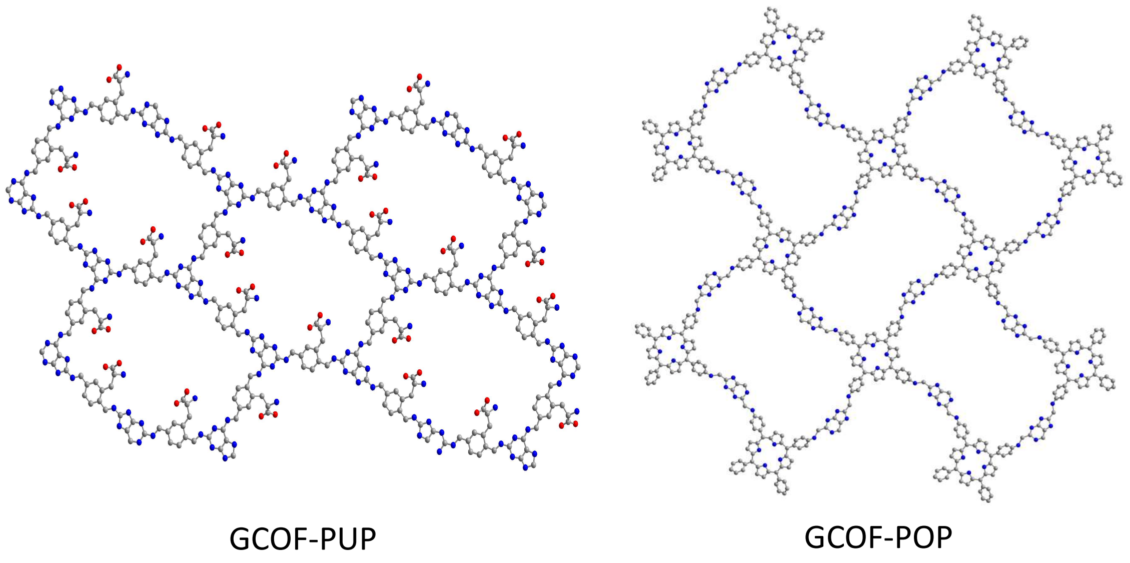

Supplementary Figure 8 | Two representatives of the generated Bio-COFs. The two materials were generated using purine/phenylalanine and porphyrin/purine molecules, respectively. (C, grey; O, red; N, blue, B, pink. The H atoms are omitted for clarity).

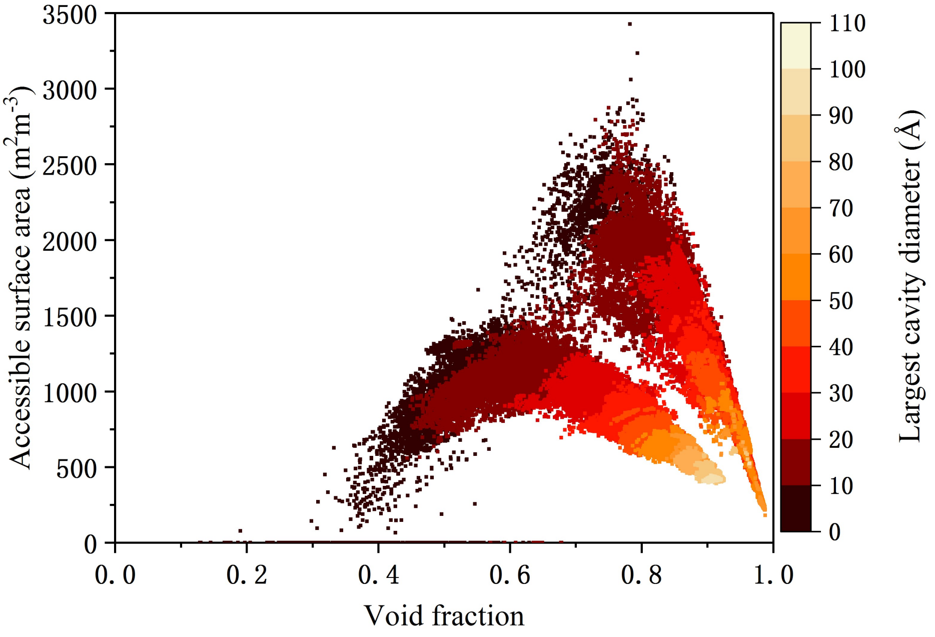

Supplementary Figure 9 | Relationship between the accessible surface area and void fraction of the COFs in our database, colored by material largest cavity diameter.

Supplementary Table 2. Comparison of the cell parameters of 10 synthesized COFs with those of the built structures and the built structures after optimization. The cell lengths and angles are in units of Å and degree, respectively.

	Material	Structure type	*a*	*b*	*c*	*α*	*β*	*γ*
2D	BLP-2H[7]	Built	15.31	15.31	3.40	90.00	90.00	120.00
		Built-Optimized	15.25	15.25	3.51	90.00	90.00	120.00
		Exp.	15.29	15.29	3.46	90.00	90.00	120.00
	COF-5[8]	Built	30.30	30.29	3.40	90.00	90.00	119.98
		Built-Optimized	30.12	30.12	3.54	90.00	90.00	120.00
		Exp.	29.70	29.70	3.46	90.00	90.00	120.00
	COF-6[9]	Built	15.10	15.10	3.40	90.00	90.00	119.99
		Built-Optimized	15.05	15.05	3.53	90.00	90.00	120.00
		Exp.	15.09	15.09	3.60	90.00	90.00	120.00
	COF-10[9]	Built	37.87	37.85	3.40	90.00	90.00	119.98
		Built-Optimized	37.74	37.74	3.54	90.00	90.00	120.00
		Exp.	37.81	37.81	3.48	90.00	90.00	120.00
	COF-LZU1[10]	Built	24.18	24.18	3.40	90.00	90.00	120.00
		Built-optimized	22.56	22.56	3.53	90.00	90.00	120.00
		Exp.	22.04	22.04	3.73	90.00	90.00	120.00
	Pc-PBBA-COF[11]	Built	23.06	23.04	3.40	90.00	90.00	90.00
		Built-optimized	23.11	23.03	3.53	90.00	90.00	90.00
		Exp.	22.85	22.85	3.34	90.00	90.00	90.00
3D	COF-102[12]	Built	27.25	27.25	27.25	90.00	90.00	90.00
		Built-Optimized	27.27	27.27	27.27	90.00	90.00	90.00
		Exp.	27.18	27.18	27.18	90.00	90.00	90.00
	COF-105[12]	Exp.	44.89	44.89	44.89	90.00	90.00	90.00
		Built	43.78	43.78	43.78	90.00	90.00	90.00
		Built-optimized	44.89	44.89	44.89	90.00	90.00	90.00
	COF-108[12]	Built	28.40	28.40	28.40	90.00	90.00	90.00
		Built-Optimized	28.40	28.40	28.40	90.00	90.00	90.00
		Exp.	28.40	28.40	28.40	90.00	90.00	90.00
	COF-202[13]	Built	30.10	30.10	30.10	90.00	90.00	90.00
		Built-Optimized	29.83	29.83	29.83	90.00	90.00	90.00
		Exp.	30.11	30.11	30.11	90.00	90.00	90.00

Supplementary Table 3. Comparison of some common structural descriptors calculated for the synthesized COFs, the built structures and the built structures after molecular mechanics optimization.

	**Material**	**Structure type**	ρ_{crys} **(g/cm^3)**	**PLD (Å)**	**LCD (Å)**	S_{acc} **(m^2/g)**	ϕ
2D	BLP-2H-AA	Built	0.92	8.9	9.4	1119	0.22
		Built-Optimized	0.90	9.1	9.6	1163	0.23
		Exp.	0.90	9.0	9.5	1141	0.22
	COF-5	Built	0.57	23.9	24.1	1748	0.55
		Built-Optimized	0.56	23.8	24.1	1805	0.55
		Exp.	0.58	23.4	23.7	1707	0.54
	COF-6	Built	1.05	8.6	9.0	1011	0.23
		Built-Optimized	1.02	8.6	9.2	1152	0.24
		Exp.	1.00	8.6	9.2	1128	0.24
	COF-10	Built	0.46	31.4	31.6	1899	0.63
		Built-Optimized	0.44	31.4	31.6	1967	0.63
		Exp.	0.45	31.3	31.5	1949	0.63
	COF-LZU1	Built	0.52	16.8	17.1	2344	0.47
		Built-Optimized	0.58	16.2	16.6	2166	0.45
		Exp.	0.57	15.6	16.0	2172	0.44
	Pc-PBBA-COF	Built	0.76	16.7	17.0	1407	0.43
		Built-Optimized	0.73	16.8	17.1	1470	0.43
		Exp.	0.79	16.6	16.8	1391	0.42
3D	COF-102	Built	0.42	8.0	9.0	5102	0.53
		Built-Optimized	0.42	8.0	9.1	5185	0.53
		Exp.	0.42	8.0	9.0	5083	0.53
	COF-105	Built	0.18	16.1	18.8	6646	0.80
		Built-Optimized	0.19	15.6	18.4	6332	0.79
		Exp.	0.18	16.1	18.8	6644	0.80
	COF-108	Built	0.17	19.1	27.5	6425	0.81
		Built-Optimized	0.17	18.9	27.2	6437	0.81
		Exp.	0.17	19.0	27.5	6387	0.81
	COF-202	Built	0.52	5.3	9.8	4271	0.40
		Built-Optimized	0.54	5.6	9.9	4108	0.41
		Exp.	0.52	5.4	9.9	4254	0.42

Supplementary Table 4. Comparison of the cell parameters of the built structures and the built-optimized structures of 8 generated functionalized 2D-COFs. These COFs are composed of different center and linker GSUs and modified using different GSUs of the functional groups. The cell lengths and angles are in units of Å and degree, respectively.

**Material**	**Structure type**	**a**	**b**	**c**	**α**	**β**	**γ**
GCOF1-F	Built	36.86	36.86	3.60	90.00	90.00	120.00
	Built-Optimized	36.69	36.69	3.59	90.00	90.00	120.00
GCOF2-Cl	Built	44.43	44.43	3.65	90.00	90.00	120.00
	Built-Optimized	45.43	45.43	3.66	90.00	90.00	120.00
GCOF3-Br	Built	44.43	44.43	3.60	90.00	90.00	120.00
	Built-Optimized	45.38	45.38	3.60	90.00	90.00	120.00
GCOF4-CH_3	Built	28.51	28.51	3.88	90.00	90.00	120.00
	Built-Optimized	29.01	29.01	4.03	90.00	90.00	120.00
GCOF5-NH_2	Built	25.10	25.10	4.60	90.00	90.00	120.00
	Built-Optimized	25.81	25.81	4.60	90.00	90.00	120.00
GCOF6-OH	Built	48.04	48.04	3.50	90.00	90.00	120.00
	Built-Optimized	48.71	48.71	3.55	90.00	90.00	120.00
GCOF7-COOH	Built	39.00	39.00	3.50	90.00	90.00	120.00
	Built-Optimized	38.20	38.20	3.54	90.00	90.00	120.00
GCOF8-NO_2	Built	15.00	15.00	3.80	90.00	90.00	120.00
	Built-Optimized	14.70	14.70	3.90	90.00	90.0	120.0

Supplementary Note 2

Materials. Tris(4-formylphenyl)amine (TFPA), tris(4-aminolphenyl)benzene (TAPB) and anhydrous solvents were obtained from commercial suppliers and used without further purification. 4,4',4'',4'''-(Ethene-1,1,2,2-tetrayl) tetraaniline (ETTA), 1,3,5-trimethyl-2,4,6-tris(4-formylphenyl)benzene (TTFB) and 1,3,5-tris(4-formylphenyl)benzene (TFPB) were synthesized according to the literature.

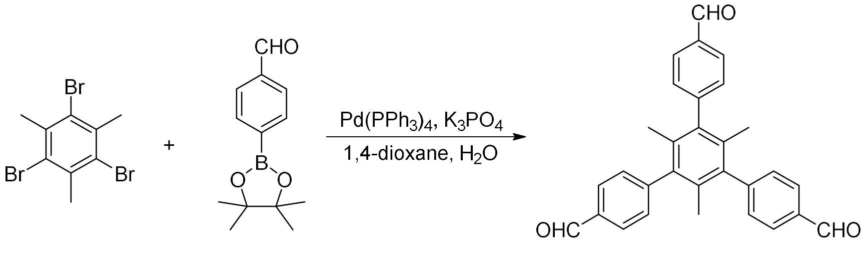

Synthesis of 1,3,5-Trimethyl-2,4,6-tris(4-formylphenyl)benzene (**TTFB**). 2,4,6-tribromomesitylene (356 mg, 1.00 mmol), 4-(4,4,5,5-tetramethyl-1,3,2-dioxaborolan-2-yl)benzaldehyde (928 mg, 4 mmol), palladium tetrakis(triphenyl phosphine) ($Pd(PPh_3)_4$) (58 mg, 0.05 mmol) and potassium phosphate (1.27 g, 6 mmol) in a mixed solvent of 1,4-dioxane (60 mL) and distilled water (5 mL) were stirred at 100 °C under an N_2 atmosphere for 24 h. After being cooled to room temperature, the solvent was removed under vacuum and the residue was purified by flash column chromatography (petroleum ether/ethyl acetate/dichloromethane (7:1:0.6); silica gel, 300-400 mesh) to give TTFB as a white solid (287 mg, 66%). ^1H NMR (500 MHz, $CDCl_3$) δ 10.07 (s, 3H), 7.98 (d, J = 10 Hz, 6H), 7.41 (d, J = 10 Hz, 6H), 1.69 (s, 9H). ^{13}C NMR (126 MHz, $CDCl_3$) δ 191.99, 148.35, 139.17, 135.28, 132.83, 130.29, 130.26, 19.48. Anal. Calcd for $C_{30}H_{24}O_3$: C, 83.31; H, 5.59. Found: C, 83.17; H, 5.67. IR (cm^{-1}): 3038, 2960, 2918, 2826, 2724, 1697, 1602, 1565, 1373, 1302, 1282, 1210, 1167, 1102, 1016, 958, 842, 721.

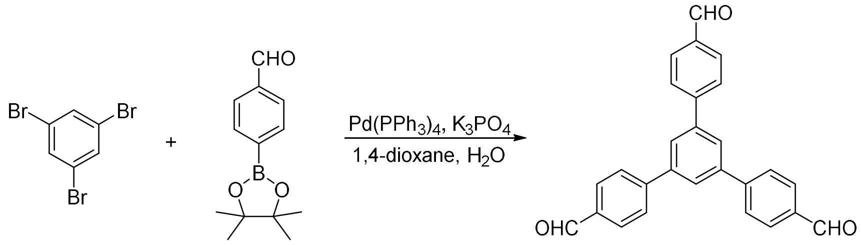

Synthesis of 1,3,5-tris(4-formylphenyl)benzene (TFPB)[14]. A mixture of 4-(4,4,5,5-tetramethyl-1,3,2-dioxaborolan-2-yl)benzaldehyde (4.64 g, 20 mmol), 1,3,5-tribromobenzene (1.57 g, 5 mmol), $Pd(PPh_3)_4$ (288.9 mg, 0.25 mmol), potassium phosphate (7.43 g, 35 mmol), 1,4-dioxane (80 mL) and distilled water (8 mL) was stirred at 90 °C under an N_2 atmosphere for 24 h. After being

cooled to room temperature, the solvent was removed under vacuum and the residue was purified by flash column chromatography (petroleum ether/ethyl acetate/dichloromethane (10/1; 4/1); silica gel, 300-400 mesh) to give TFPB as a white solid (1.7 g, 87%). ^{1}H NMR (400 MHz, $CDCl_3$) : δ 10.11 (s, 3H), 8.03 (d, J = 8.2 Hz, 6H), 7.91 (s, 3H), 7.88 (d, J = 8.2 Hz, 6H). IR (cm^{-1}): 3041, 2844, 2716, 1685, 1601, 1567, 1384, 1212, 1168, 1118, 872, 855, 813, 792.

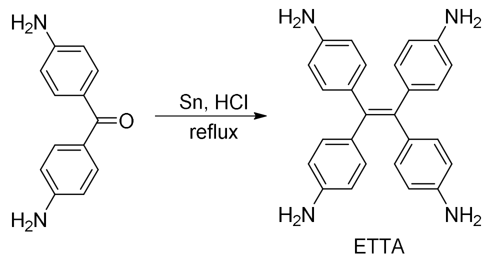

Synthesis of 4,4',4',4'''-(ethene-1,1,2,2-tetrayl) tetraaniline (ETTA)[15]. A solution of 4, 4'-diaminobenzophenone (0.80 g) in concentrated hydrochloric acid (36 mL) was heated under reflux for 24 h with granulated tin. After being cooled to room temperature, saturated $NaHCO_3$ was slowly added until pH = 7. The organic phase was collected and dried over anhydrous Na_2SO_4. After removal of solvent, the residue was purified by chromatography (CH_2Cl_2/EA (10:1); silica gel, 300-400 mesh) to give ETTA as a light green solid (0.13 g, 63%). ^{1}H NMR (400 MHz, DMSO-d_6) δ 6.56 (d, J = 8.2 Hz, 8H), 6.25 (d, J = 8.2 Hz, 8H,), 4.86 (s, 8H). IR (cm^{-1}): 3437, 3353, 3204, 3019, 2918, 2848, 1614, 1564, 1510, 1427, 1273, 1175, 1122, 1056, 1006, 978, 936, 829, 784, 745.

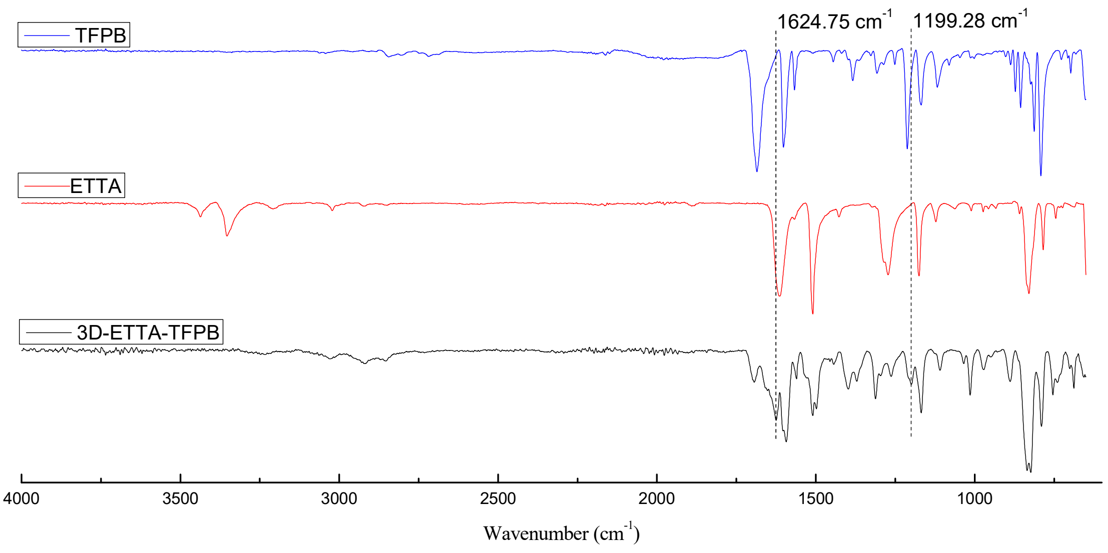

Supplementary Figure 10 | FT-IR spectra for the comparison between the starting materials (TFPB and ETTA) and **3D-ETTA-TFPB**.

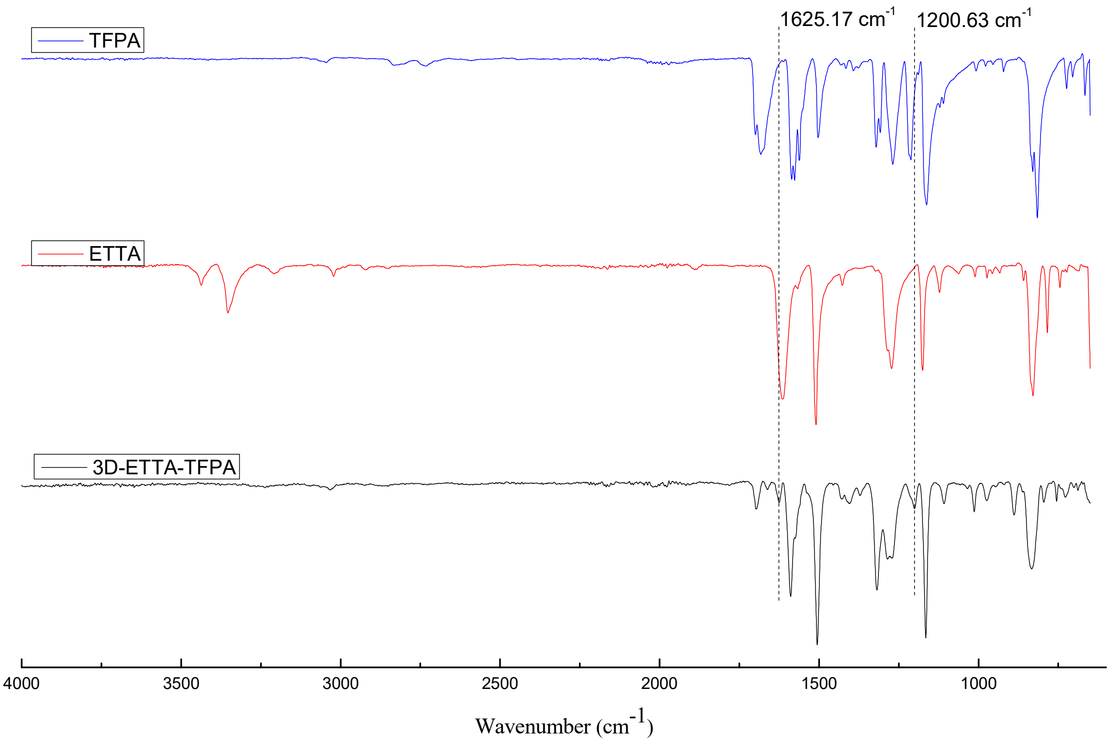

Supplementary Figure 11 | FT-IR spectra for the comparison between the starting materials (TFPA and ETTA) and **3D-ETTA-TFPA**.

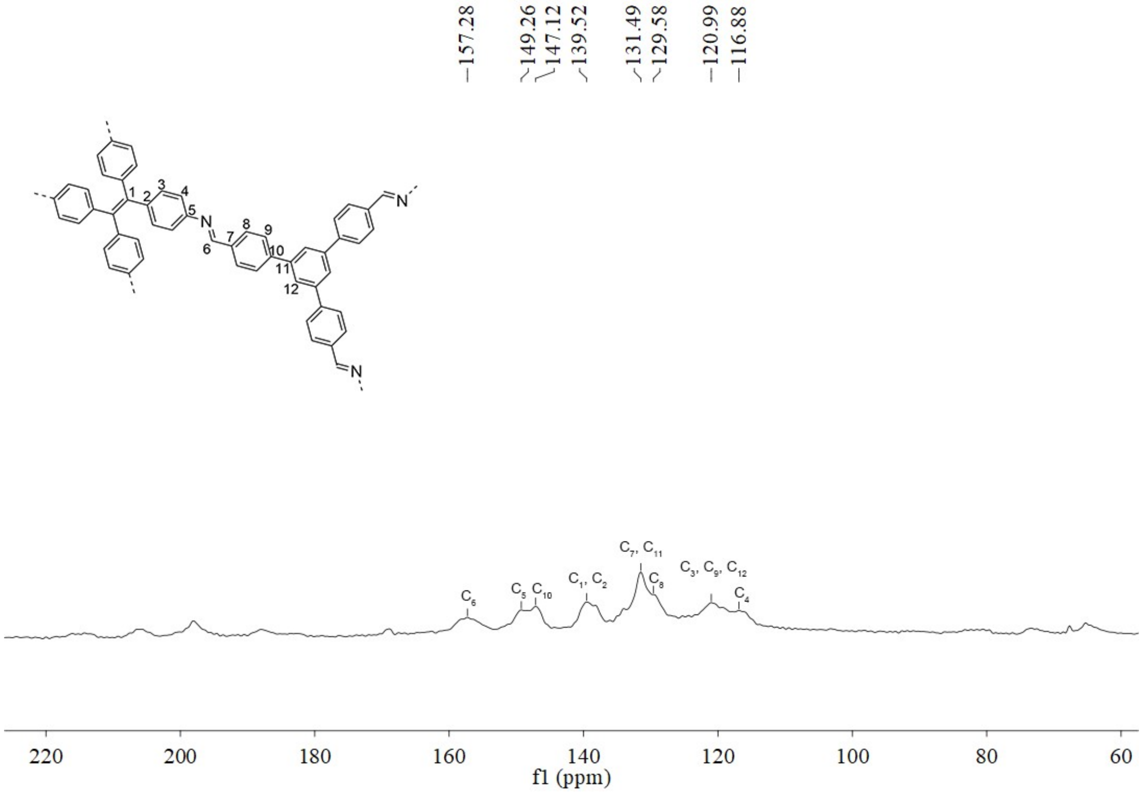

Supplementary Figure 12 | Solid-state ^{13}C CP/MAS NMR spectrum of **3D-ETTA-TFPB**.

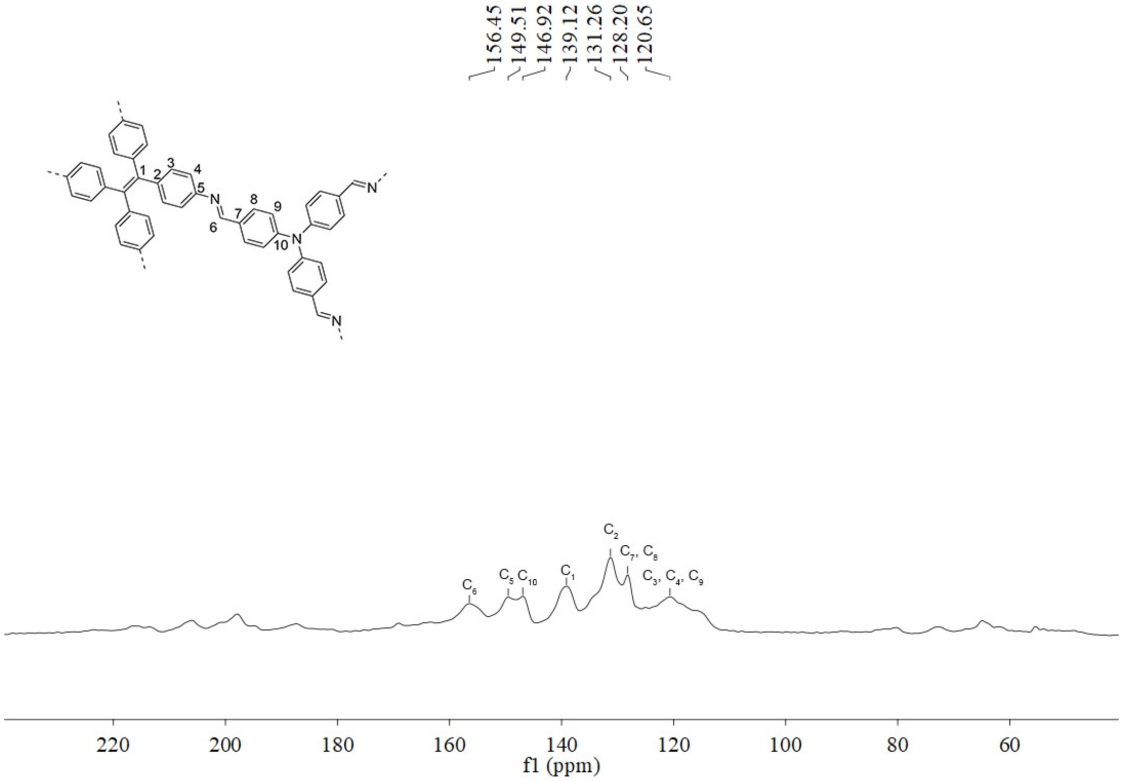

Supplementary Figure 13 | Solid-state ^{13}C CP/MAS NMR spectrum of **3D-ETTA-TFPA**.

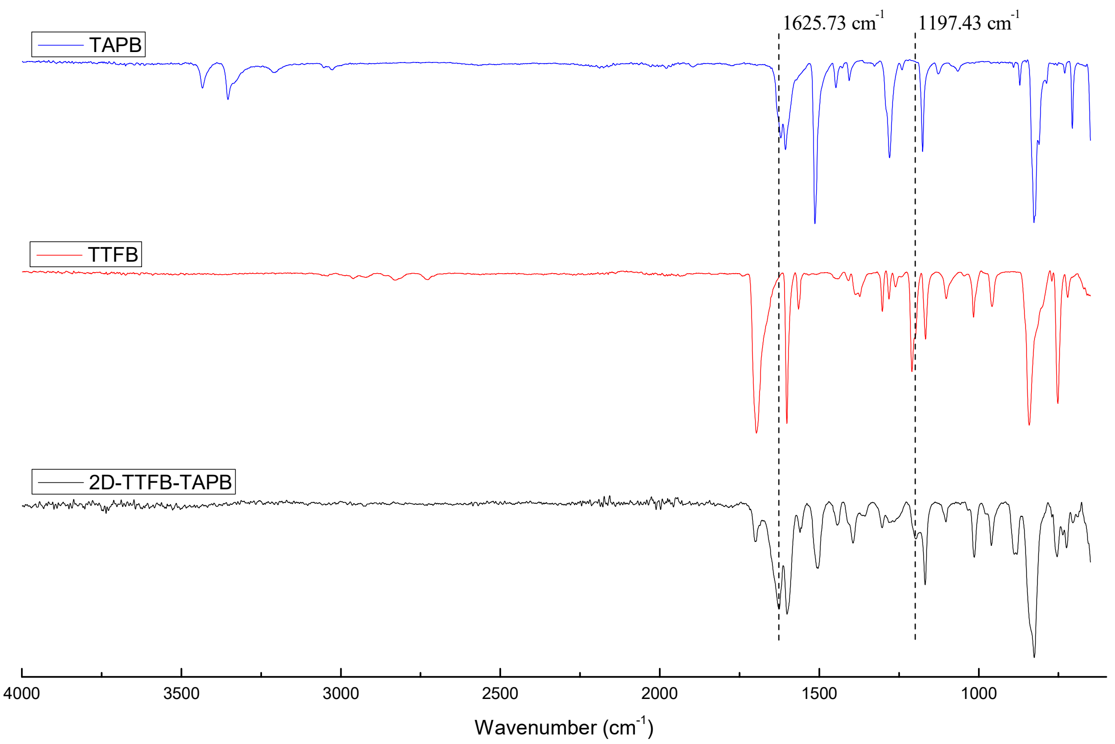

Supplementary Figure 14 | FT-IR spectra for the comparison between the starting materials (TAPB and TTFB) and **2D-TTFB-TAPB**.

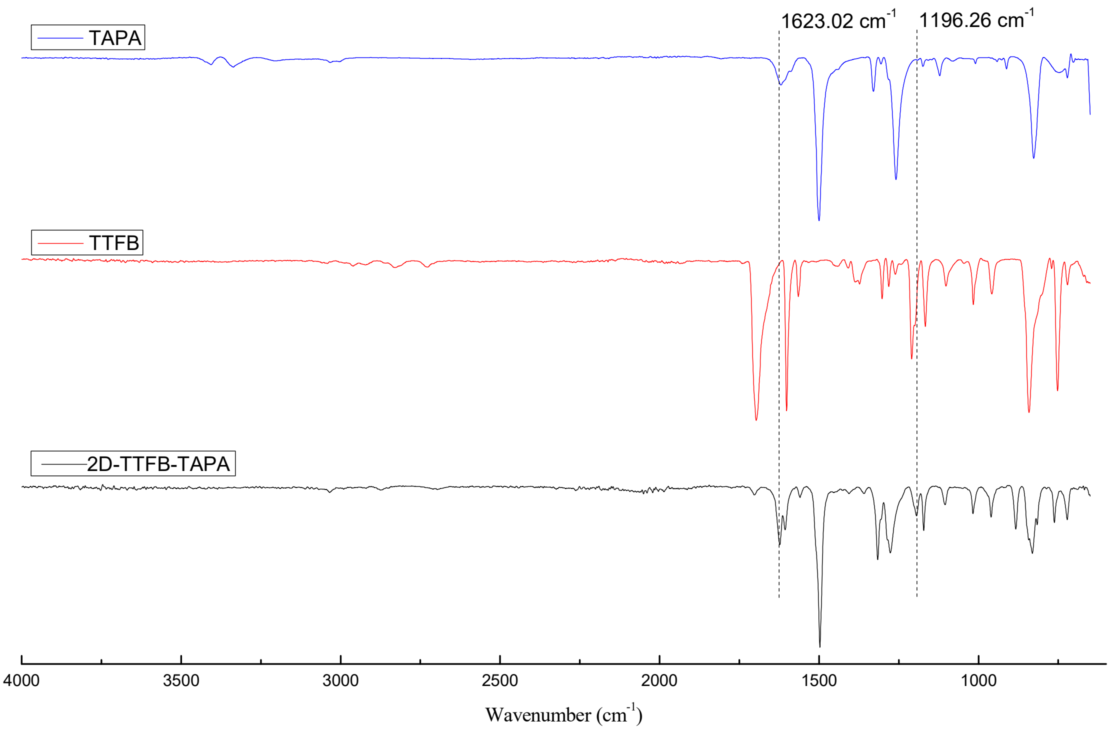

Supplementary Figure 15 | FT-IR spectra for the comparison between the starting materials (TAPA and TTFB) and **2D-TTFB-TAPA**.

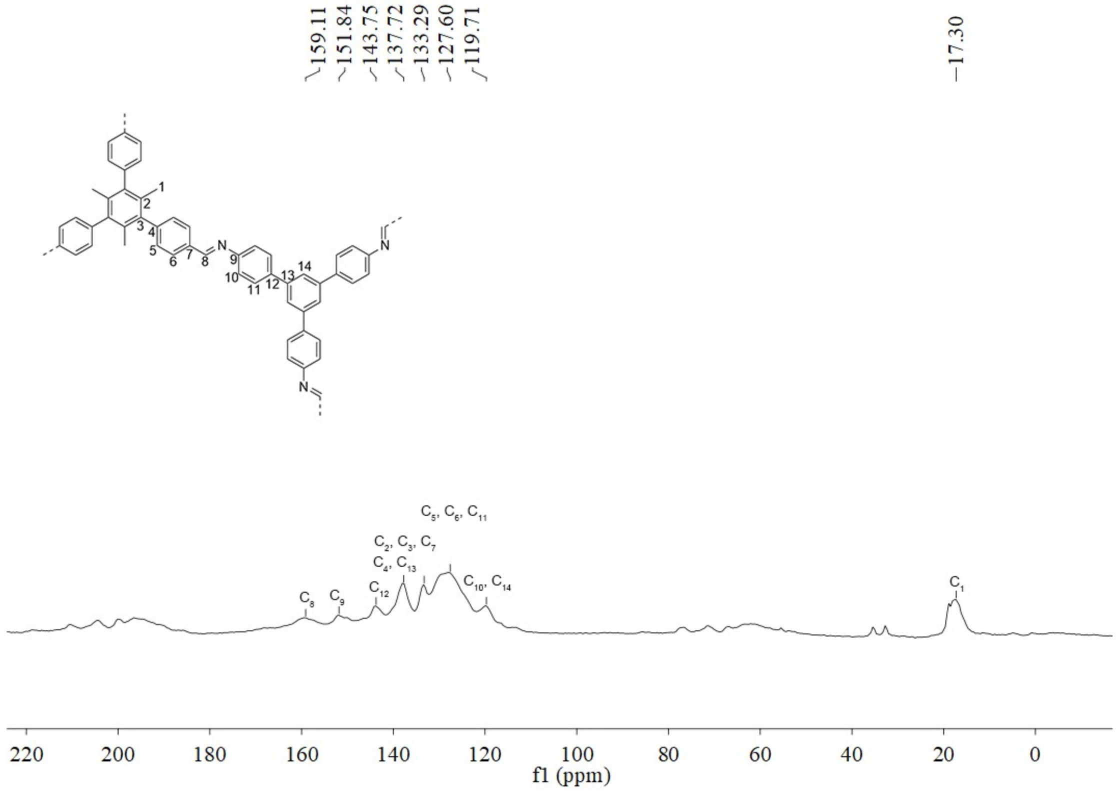

Supplementary Figure 16| Solid-state ^{13}C CP/MAS NMR spectrum of **2D-TTFB-TAPB**.

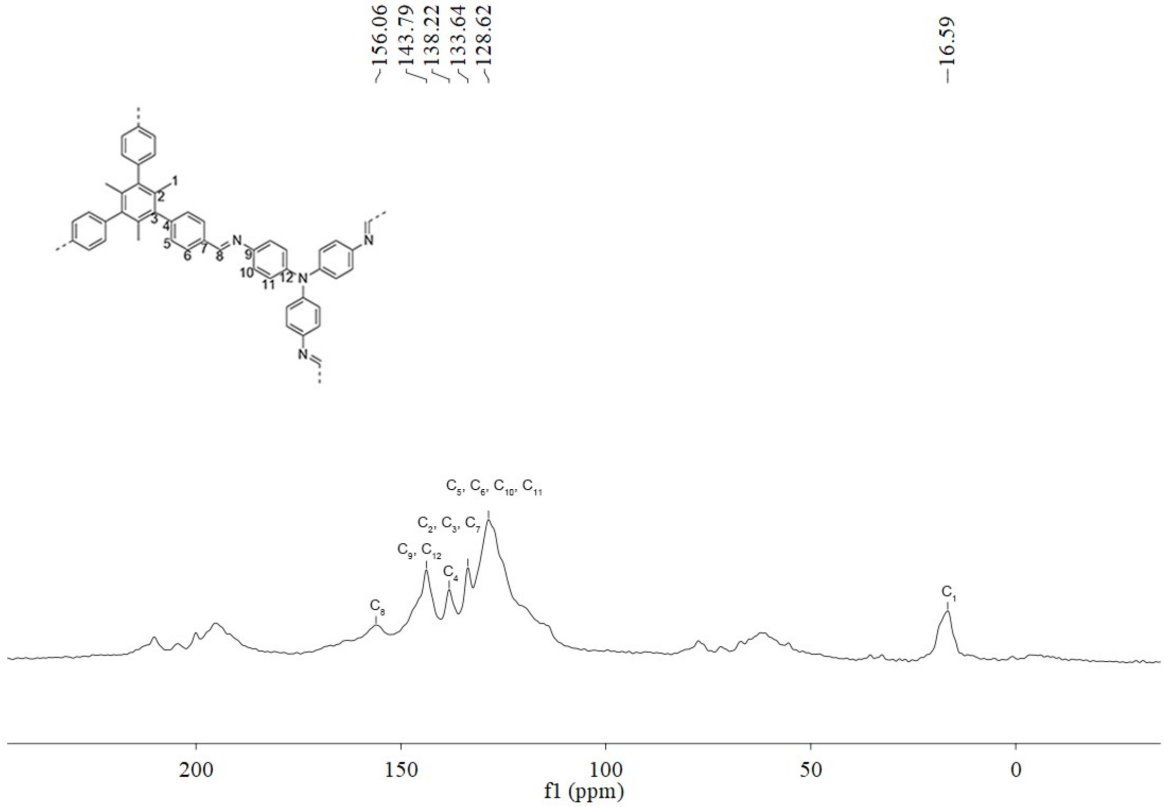

Supplementary Figure 17 | Solid-state ^{13}C CP/MAS NMR spectrum of **2D-TTFB-TAPA**.

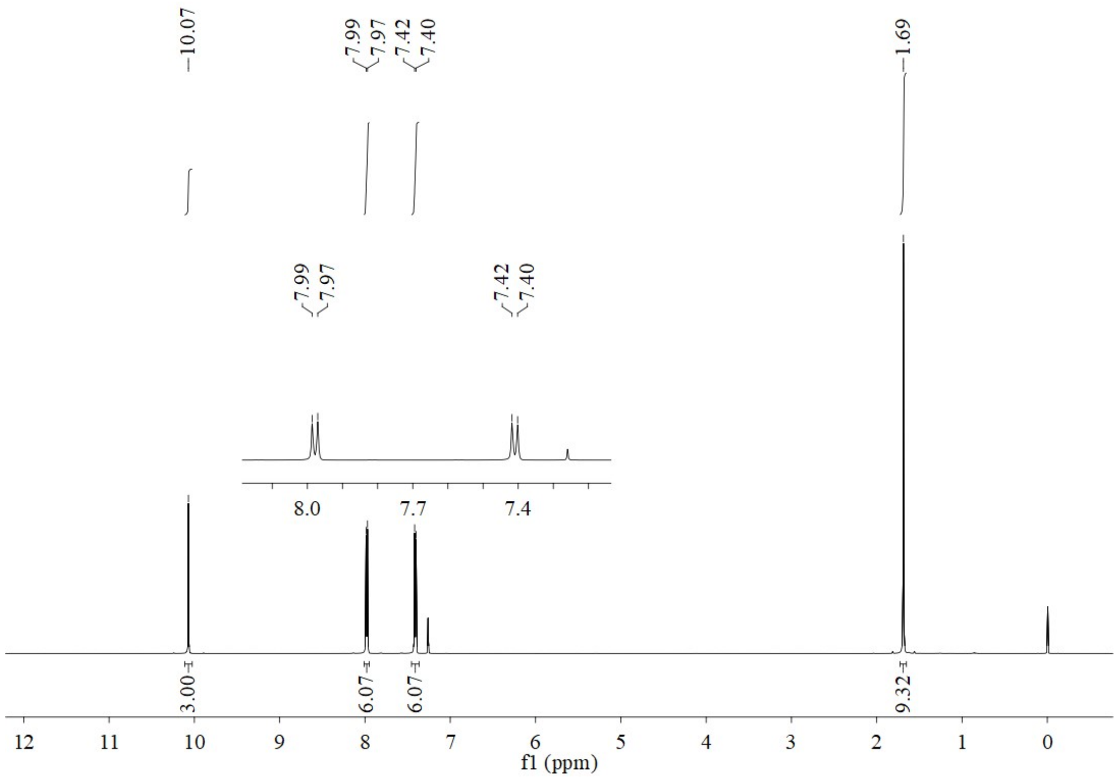

Supplementary Figure 18 | ^{1}H NMR (500 MHz, $CDCl_3$) spectrum of TTFB.

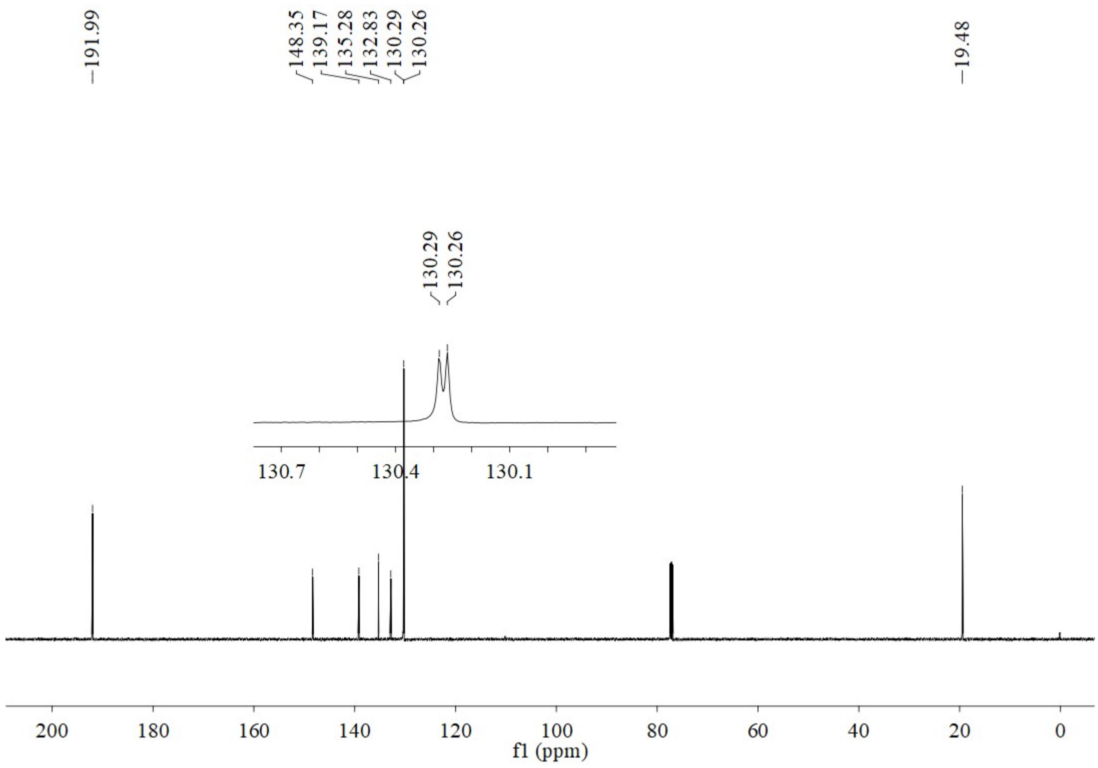

Supplementary Figure 19 | ^{13}C NMR (125 MHz, $CDCl_3$) spectrum of TTFB.

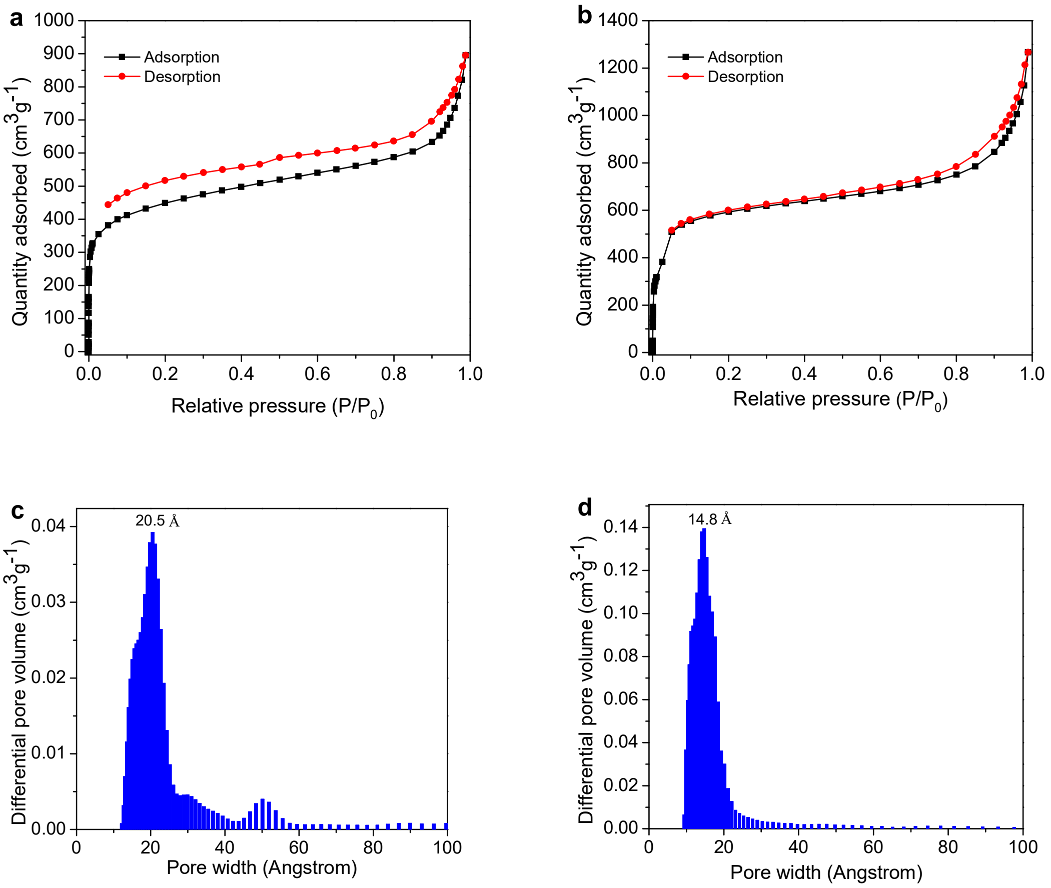

Supplementary Figure 20 | Gas adsorption. N_2 adsorption isotherms (77 K) of (**a**) **2D-TTFB-TAPB** and (**b**) **2D-TTFB-TAPA**. Pore size distribution profiles of (**c**) **2D-TTFB-TAPB** and (**d**) **2D-TTFB-TAPA**.

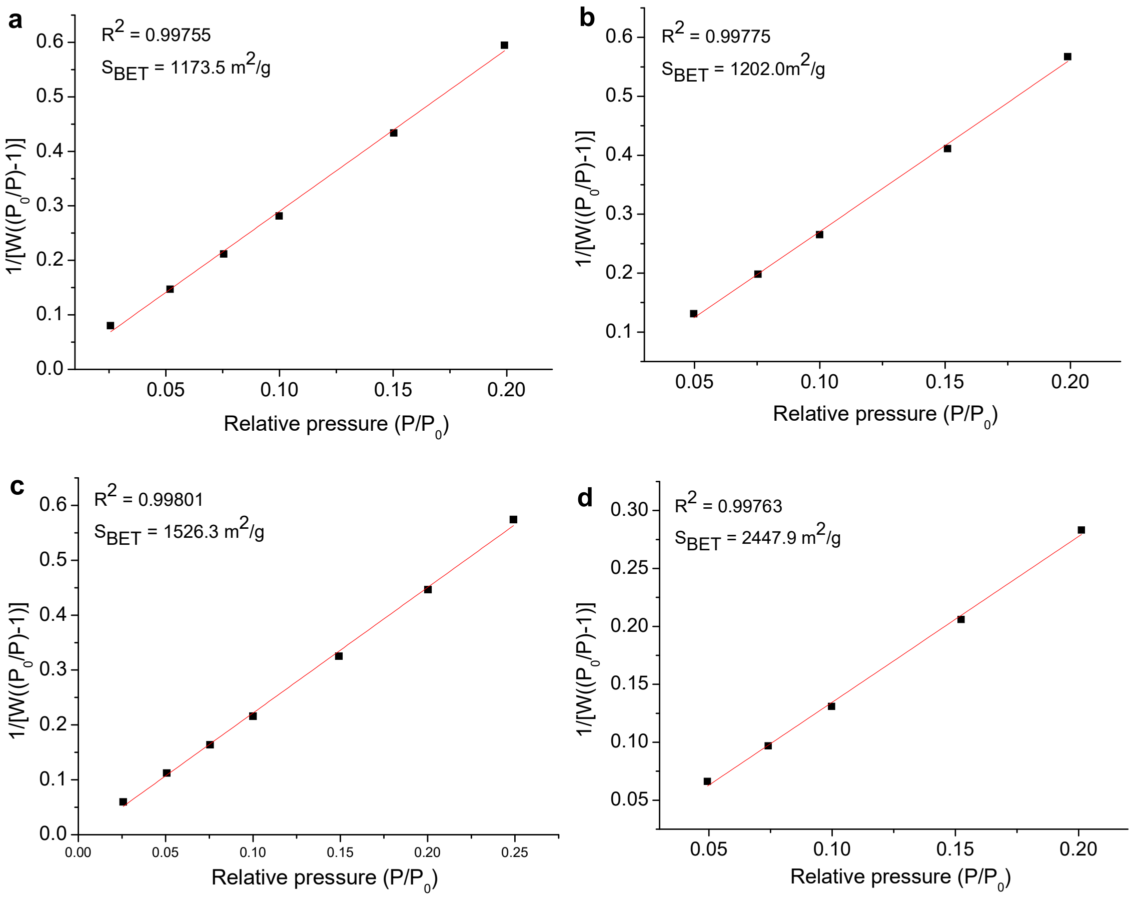

Supplementary Figure 21 | BET surface areas. BET surface area plots for (**a**) **3D-ETTA-TFPB**, (**b**) **3D-ETTA-TFPA**, (**c**) **2D-TTFB-TAPB**, and (**d**) **2D-TTFB-TAPA**.

Supplementary Table 5. Unit cell parameters and fractional atomic coordinates for 3D-ETTA-TFPB with **ffc** topology.

Space group	P112		
Calculated unit cell	a = 46.1582 Å, b = 45.6774 Å, c = 20.9113 Å, $\alpha = \beta$ = 90°, γ = 120°		
Measured unit cell	a = 46.1341 Å, b = 45.6374 Å, c = 20.8920 Å, $\alpha = \beta$ = 90°, γ = 120°		
Pawley refinement	R_p = 2.51%, R_{wp} = 3.53%		
Atom	x	y	z
C1	0.50472	0.97643	0.09176
C2	0.50462	0.97634	0.95156
C3	0.53821	0.98356	0.10427
C4	0.54288	0.96152	0.14546
C5	0.51389	0.93297	0.17290
C6	0.48053	0.92519	0.15800
C7	0.47578	0.94717	0.11685
C8	0.47622	0.94358	0.93943
C9	0.48038	0.92103	0.89876
C10	0.51286	0.93193	0.87139
C11	0.54136	0.96408	0.88578
C12	0.53729	0.98668	0.92643
C13	0.97375	0.50263	0.05120
C14	0.97146	0.49726	0.90378
C15	0.93897	0.47708	0.05084
C16	0.91417	0.48304	0.07968
C17	0.92664	0.51799	0.11089
C18	0.96384	0.54210	0.11467
C19	0.97889	0.52961	0.08326
C20	0.97013	0.52741	0.89137
C21	0.94424	0.52500	0.85106
C22	0.92070	0.49280	0.82473
C23	0.92154	0.46241	0.83950
C24	0.94738	0.46468	0.87963
C25	0.52164	0.54797	0.52020
C26	0.51658	0.51464	0.51615
C27	0.45435	0.48848	0.51116
C28	0.51104	0.55786	0.57681
C29	0.51504	0.59076	0.57976
C30	0.53002	0.61311	0.52667
C31	0.54137	0.60359	0.47045
C32	0.53702	0.57059	0.46721
C33	0.44897	0.50210	0.45334

C34	0.42009	0.50546	0.44841
C35	0.39708	0.49476	0.50136
C36	0.40220	0.48077	0.55858
C37	0.43124	0.47767	0.56378
C38	0.46647	0.19702	0.57955
C39	0.47089	0.16831	0.58189
C40	0.47000	0.15219	0.52299
C41	0.46518	0.16395	0.46307
C42	0.46142	0.19305	0.46320
C43	0.46186	0.21020	0.52075
C44	0.45852	0.23943	0.51892
C45	0.42902	0.23748	0.49108
C46	0.51030	0.17320	0.66527
C47	0.47691	0.15626	0.63958
C48	0.46419	0.14696	0.40615
C49	0.43220	0.12209	0.38105
C50	0.42643	0.26716	0.48708
C51	0.45329	0.29802	0.51171
C52	0.48256	0.30042	0.54066
C53	0.48518	0.27078	0.54401
C54	0.43125	0.10332	0.32605
C55	0.46221	0.10994	0.29802
C56	0.49420	0.13544	0.32155
C57	0.49521	0.15429	0.37643
C58	0.45001	0.12671	0.66917
C59	0.45703	0.11303	0.72418
C60	0.49069	0.12941	0.74800
C61	0.51725	0.15953	0.72038
N62	0.46870	0.35674	0.53391
C63	0.44989	0.32659	0.50416
C64	0.50024	0.11574	0.79819
N65	0.48035	0.08566	0.82735
N66	0.48522	0.08816	0.21749
C67	0.45934	0.08851	0.24753
C68	0.21999	0.51267	0.53222
C69	0.18815	0.51049	0.53467
C70	0.16969	0.50348	0.47667
C71	0.18177	0.49895	0.41679
C72	0.21380	0.50122	0.41755
C73	0.23345	0.50789	0.47421
C74	0.26479	0.50927	0.47287
C75	0.29184	0.53516	0.43592
C76	0.18258	0.54874	0.60921

C77	0.17471	0.51510	0.59169
C78	0.16281	0.49274	0.35972
C79	0.15469	0.46333	0.32148
C80	0.32308	0.53606	0.43399
C81	0.32653	0.51123	0.46949
C82	0.29998	0.48544	0.50684
C83	0.26872	0.48442	0.50829
C84	0.13567	0.45723	0.26447
C85	0.12489	0.48058	0.24734
C86	0.13258	0.50994	0.28459
C87	0.15185	0.51606	0.34107
C88	0.15235	0.48581	0.62855
C89	0.13630	0.49016	0.68271
C90	0.14323	0.52365	0.69828
C91	0.16652	0.55301	0.66348
N92	0.36846	0.49625	0.50236
C93	0.35776	0.51330	0.46510
C94	0.12579	0.52950	0.74666
N95	0.10060	0.50537	0.78234
N96	0.08824	0.48767	0.17220
C97	0.10670	0.47366	0.19033
H98	0.56033	1.00649	0.08135
H99	0.56921	0.96659	0.15624
H100	0.45829	0.90166	0.17906
H101	0.44966	0.94199	0.10364
H102	0.45104	0.93616	0.96224
H103	0.45824	0.89486	0.88829
H104	0.56684	0.97122	0.86472
H105	0.55929	1.01256	0.93921
H106	0.93068	0.45157	0.02737
H107	0.88671	0.46244	0.07884
H108	0.91575	0.53121	0.08144
H109	0.97388	0.56674	0.14254
H110	0.98952	0.55242	0.91361
H111	0.94245	0.54849	0.84012
H112	0.90166	0.43712	0.81904
H113	0.94931	0.44149	0.89278
H114	0.49963	0.53942	0.61840
H115	0.50639	0.59893	0.62388
H116	0.55356	0.62241	0.42966
H117	0.54551	0.56192	0.42353
H118	0.46772	0.50998	0.41241
H119	0.41549	0.51646	0.40317

H120	0.38314	0.47232	0.59905
H121	0.43637	0.46683	0.60867
H122	0.46669	0.20955	0.62684
H123	0.47320	0.12901	0.52276
H124	0.45791	0.20353	0.41620
H125	0.40802	0.21228	0.47251
H126	0.53065	0.19715	0.64145
H127	0.40822	0.11773	0.40520
H128	0.40334	0.26594	0.46465
H129	0.50298	0.32568	0.56027
H130	0.50813	0.27165	0.56630
H131	0.40619	0.08349	0.30529
H132	0.51806	0.14021	0.29631
H133	0.51997	0.17487	0.39694
H134	0.42368	0.11471	0.64845
H135	0.43614	0.08956	0.74831
H136	0.54353	0.17258	0.74156
H137	0.42998	0.32569	0.47066
H138	0.52679	0.13020	0.81794
H139	0.43358	0.06931	0.22841
H140	0.23565	0.51841	0.57753
H141	0.14396	0.50124	0.47666
H142	0.22483	0.49765	0.37142
H143	0.28806	0.55461	0.40867
H144	0.20137	0.57130	0.57977
H145	0.16350	0.44513	0.33698
H146	0.34462	0.55626	0.40462
H147	0.30424	0.46639	0.53443
H148	0.24688	0.46424	0.53708
H149	0.12939	0.43419	0.23375
H150	0.12326	0.52766	0.26860
H151	0.15866	0.53929	0.37164
H152	0.14781	0.45979	0.61408
H153	0.11840	0.46734	0.71264
H154	0.17234	0.57942	0.67835
H155	0.37665	0.53043	0.42778
H156	0.13241	0.55646	0.75893
H157	0.10672	0.45477	0.15471
C158	0.00000	0.50000	0.01581
C159	0.00000	0.50000	0.94107
C160	0.50000	1.00000	-0.01256
C161	0.50000	1.00000	0.05578

Supplementary Table 6. Unit cell parameters and fractional atomic coordinates for 3D-ETTA-TFPA with **ffc** topology.

Space group	P112		
Calculated unit cell	a = 41.8574 Å, b = 41.3181 Å, c = 17.8801 Å, $\alpha = \beta$ = 90°, γ = 120°		
Measured unit cell	a = 41.8782 Å, b = 41.2827 Å, c = 17.8371 Å, $\alpha = \beta$ = 90°, γ = 120°		
Pawley refinement	R_p = 4.32%, R_{wp} = 5.49%		
Atom	x	y	z
C1	0.52986	0.50305	0.09077
C2	0.53004	0.50330	0.92630
C3	0.56526	0.53534	0.08765
C4	0.59385	0.53878	0.13462
C5	0.58744	0.51000	0.18548
C6	0.55263	0.47731	0.18661
C7	0.52409	0.47377	0.13983
C8	0.54116	0.47617	0.93052
C9	0.56982	0.47896	0.88470
C10	0.58777	0.50887	0.83369
C11	0.57747	0.53648	0.83114
C12	0.54894	0.53381	0.87684
C13	0.46943	0.97371	0.05049
C14	0.46858	0.97553	0.88640
C15	0.47388	0.94951	0.09988
C16	0.44463	0.92465	0.14587
C17	0.41036	0.92324	0.14330
C18	0.40527	0.94638	0.09236
C19	0.43465	0.97154	0.04654
C20	0.45081	0.99030	0.84246
C21	0.42009	0.96664	0.79835
C22	0.40649	0.92770	0.79700
C23	0.42384	0.91293	0.84184
C24	0.45457	0.93652	0.88637
C25	0.97025	0.52235	0.56466
C26	0.00167	0.51805	0.56363
C27	0.96394	0.44872	0.55617
C28	0.94518	0.50930	0.62522
C29	0.91261	0.51136	0.62227
C30	0.90435	0.52597	0.55871
C31	0.93021	0.54061	0.49978
C32	0.96285	0.53888	0.50273
C33	0.94156	0.44335	0.49289

C34	0.90802	0.40987	0.48328
C35	0.89623	0.38081	0.53640
C36	0.91841	0.38616	0.59964
C37	0.95194	0.41978	0.60972
C38	0.25106	0.47891	0.50604
C39	0.24165	0.49367	0.44374
C40	0.31245	0.51686	0.61967
C41	0.30284	0.48509	0.57496
C42	0.30024	0.47569	0.44580
C43	0.28208	0.44341	0.40067
C44	0.20786	0.49316	0.44026
C45	0.18241	0.47839	0.49994
C46	0.19151	0.46372	0.56254
C47	0.22512	0.46372	0.56509
C48	0.29935	0.43932	0.33657
C49	0.33532	0.46747	0.31597
C50	0.33572	0.50421	0.42435
C51	0.31392	0.45961	0.59870
C52	0.33542	0.46653	0.66347
C53	0.34647	0.49916	0.70674
C54	0.33389	0.52380	0.68477
N55	0.12846	0.47510	0.55673
C56	0.14901	0.47861	0.49554
C57	0.37037	0.50790	0.76846
N58	0.38627	0.48709	0.78375
N59	0.38819	0.48865	0.23658
C60	0.35320	0.46244	0.25392
C61	0.74903	0.22199	0.47318
C62	0.74911	0.24980	0.42684
C63	0.68960	0.17632	0.58824
C64	0.70630	0.16502	0.53352
C65	0.70465	0.16927	0.40416
C66	0.72452	0.16299	0.34775
C67	0.77800	0.28710	0.42987
C68	0.80784	0.29789	0.47978
C69	0.80777	0.27025	0.52660
C70	0.77889	0.23293	0.52312
C71	0.70681	0.14407	0.28140
C72	0.66886	0.13121	0.26978
C73	0.64957	0.13897	0.32555
C74	0.66717	0.15765	0.39171
C75	0.70591	0.13094	0.54350
C76	0.68716	0.10747	0.60432

C77	0.66805	0.11742	0.65633
C78	0.67059	0.15275	0.64884
N79	0.86512	0.34721	0.52988
C80	0.83677	0.33525	0.48097
C81	0.64565	0.09141	0.71098
N82	0.62126	0.09641	0.75201
N83	0.61480	0.09962	0.19372
C84	0.65061	0.11027	0.20560
N85	0.72081	0.18577	0.47030
N86	0.28414	0.47956	0.50913
C87	0.64702	0.49983	0.36052
H88	0.57025	0.55674	0.05197
H89	0.61905	0.56249	0.13214
H90	0.54779	0.45604	0.22250
H91	0.49892	0.44982	0.14246
H92	0.52813	0.45398	0.96632
H93	0.57712	0.45886	0.88820
H94	0.59051	0.55851	0.79503
H95	0.54182	0.55414	0.87325
H96	0.49858	0.95009	0.10327
H97	0.44853	0.90776	0.18208
H98	0.38052	0.94553	0.08926
H99	0.43066	0.98861	0.01100
H100	0.46028	1.01845	0.84194
H101	0.40827	0.97826	0.76635
H102	0.41422	0.88479	0.84162
H103	0.46682	0.92516	0.91820
H104	0.95029	0.49785	0.67124
H105	0.89445	0.50149	0.66600
H106	0.92541	0.55257	0.45438
H107	0.98090	0.54927	0.45875
H108	0.94974	0.46365	0.45279
H109	0.89269	0.40685	0.43675
H110	0.91018	0.36534	0.63856
H111	0.96762	0.42295	0.65604
H112	0.25969	0.50523	0.40016
H113	0.30426	0.53540	0.60398
H114	0.25622	0.42263	0.41507
H115	0.20222	0.50402	0.39393
H116	0.17357	0.45271	0.60656
H117	0.23061	0.45240	0.61072
H118	0.28551	0.41542	0.30546
H119	0.30661	0.43601	0.56811

H120	0.34333	0.44764	0.67828
H121	0.34073	0.54736	0.71527
H122	0.14060	0.48263	0.44428
H123	0.37669	0.53094	0.80015
H124	0.33990	0.43840	0.22317
H125	0.72779	0.24298	0.39057
H126	0.69044	0.20138	0.58283
H127	0.75186	0.17182	0.35561
H128	0.77696	0.30628	0.39518
H129	0.82892	0.27706	0.56346
H130	0.77979	0.21354	0.55722
H131	0.72183	0.13947	0.24195
H132	0.62223	0.13064	0.31903
H133	0.65251	0.16259	0.43136
H134	0.71880	0.12289	0.50540
H135	0.68685	0.08261	0.60904
H136	0.65796	0.16125	0.68674
H137	0.83622	0.35362	0.44339
H138	0.64675	0.06738	0.71707
H139	0.66431	0.10244	0.16902
H140	0.62117	0.47862	0.34720
H141	0.65086	0.47194	1.45562
C142	0.50000	0.50000	0.04821
C143	0.50000	0.50000	0.96863
C144	0.50000	1.00000	0.00815
C145	0.50000	1.00000	0.92862

Supplementary Table 7. Unit cell parameters and fractional atomic coordinates for 2D-TTFB-TAPB with **hcb** topology.

Space group	P1		
Calculated unit cell	a = 25.8003 Å, b = 25.4770 Å, c = 4.7219 Å, $\alpha = \beta$ = 90°, γ = 120°		
Measured unit cell	a = 25.9255 Å, b = 25.3467 Å, c = 4.8289 Å, $\alpha = \beta$ = 90°, γ = 120°		
Pawley refinement	R_p = 4.54%, R_{wp} = 5.54%		
Atom	x	y	z
C1	-4.78058	-4.47435	0.22047
C2	-4.71781	-4.43995	0.20427
C3	-4.68949	-4.37632	0.20998
C4	-4.72297	-4.34698	0.23689
C5	-4.78552	-4.38198	0.24792
C6	-4.81467	-4.44555	0.24850
C7	-4.68132	-4.47001	0.21777
C8	-4.69271	-4.28049	0.28366
C9	-4.87973	-4.48085	0.30974
C10	-4.91788	-4.53475	0.16401
C11	-4.97939	-4.56671	0.22318
C12	-5.00332	-4.54517	0.43131
C13	-4.96501	-4.49219	0.57883
C14	-4.90399	-4.46041	0.52005
C15	-4.69501	-4.52100	0.05080
C16	-4.66098	-4.54982	0.07470
C17	-4.61293	-4.52827	0.26515
C18	-4.60001	-4.47769	0.43503
C19	-4.63353	-4.44881	0.40983
C20	-4.71465	-4.25665	0.48954
C21	-4.68514	-4.19457	0.54563
C22	-4.63234	-4.15487	0.39889
C23	-4.61109	-4.17842	0.19001
C24	-4.64072	-4.24063	0.13329
N25	-5.06565	-4.57490	0.50373
N26	-4.58141	-4.56128	0.29822
N27	-4.59899	-4.09079	0.45393
C28	-4.52887	-4.53927	0.41411
C29	-4.50331	-4.57797	0.48527
C30	-4.46104	-4.55923	0.70237
C31	-4.43933	-4.59645	0.79621
C32	-4.45861	-4.65383	0.67294
C33	-4.49690	-4.66939	0.43499

C34	-4.52096	-4.63329	0.34982
C35	-4.43888	-4.69754	0.77723
C36	-4.47903	-4.76141	0.77394
C37	-4.45597	-4.80191	0.78608
C38	-4.39296	-4.77802	0.79578
C39	-4.35369	-4.71450	0.79493
C40	-4.37804	-4.67654	0.84551
C41	-4.54632	-4.78654	0.77274
C42	-4.49649	-4.86896	0.75422
C43	-4.36735	-4.82001	0.83236
C44	-4.28974	-4.68852	0.69561
C45	-4.33472	-4.60975	0.87941
C46	-4.48642	-4.89964	0.53353
C47	-4.52085	-4.96248	0.50719
C48	-4.56870	-4.99600	0.69254
C49	-4.58003	-4.96562	0.90980
C50	-4.54345	-4.90297	0.94430
C51	-4.24059	-4.63706	0.81681
C52	-4.18251	-4.61575	0.71750
C53	-4.17140	-4.64477	0.49275
C54	-4.21973	-4.69514	0.36684
C55	-4.27804	-4.71566	0.46257
C56	-4.60673	-5.06230	0.66356
C57	-4.10996	-4.62206	0.38614
H58	-4.80244	-4.52332	0.23153
H59	-4.64107	-4.34975	0.20715
H60	-4.81170	-4.35958	0.26468
H61	-4.90031	-4.55144	0.00080
H62	-5.00744	-4.60767	0.10543
H63	-4.98266	-4.47554	0.74194
H64	-4.87558	-4.42008	0.64237
H65	-4.73176	-4.53830	-0.09860
H66	-4.67221	-4.58917	-0.05476
H67	-4.56569	-4.46135	0.59604
H68	-4.62335	-4.41098	0.54859
H69	-4.75394	-4.28624	0.61324
H70	-4.70394	-4.17860	0.70510
H71	-4.57101	-4.14839	0.07221
H72	-4.62317	-4.25751	-0.02916
H73	-4.50433	-4.49253	0.48258
H74	-4.44647	-4.51642	0.80917
H75	-4.40955	-4.57928	0.97281
H76	-4.50977	-4.71074	0.31902

H77	-4.55241	-4.64810	0.17543
H78	-4.56671	-4.81244	0.96811
H79	-4.56682	-4.81570	0.58399
H80	-4.55949	-4.75142	0.77899
H81	-4.36646	-4.84096	0.62832
H82	-4.39400	-4.85635	0.98687
H83	-4.32088	-4.79575	0.91448
H84	-4.31944	-4.58738	0.67011
H85	-4.29631	-4.60168	1.01383
H86	-4.35441	-4.58796	1.00811
H87	-4.45195	-4.87465	0.37896
H88	-4.51070	-4.98452	0.33751
H89	-4.61615	-4.99062	1.05851
H90	-4.55087	-4.88187	1.12599
H91	-4.24463	-4.61310	0.99383
H92	-4.14616	-4.57657	0.81977
H93	-4.21259	-4.71784	0.18980
H94	-4.31451	-4.75297	0.35275
H95	-4.64046	-5.08637	0.82309
H96	-4.10296	-4.64446	0.20802

Supplementary Table 8. Unit cell parameters and fractional atomic coordinates for 2D-TTFB-TAPA with **hcb** topology.

Space group	P1		
Calculated unit cell	a = 23.8234 Å, b = 23.1705 Å, c = 5.7528 Å, $\alpha = \beta$ = 90°, γ = 120°		
Measured unit cell	a = 24.4371 Å, b = 23.1107 Å, c = 5.6147 Å, $\alpha = \beta$ = 90°, γ = 120°		
Pawley refinement	R_p = 3.50%, R_{wp} = 4.72%		
Atom	x	y	z
N1	-0.61171	-2.15206	0.36359
C2	-0.64838	-2.11851	0.38992
C3	-0.64423	-2.22447	0.35476
C4	-0.54261	-2.11244	0.39387
C5	-0.68489	-2.12790	0.59203
C6	-0.72088	-2.09568	0.61873
C7	-0.72040	-2.05300	0.44291
C8	-0.68421	-2.04433	0.24086
C9	-0.64853	-2.07677	0.21403
C10	-0.60994	-2.25877	0.39140

C11	-0.64251	-2.32818	0.40509
C12	-0.70941	-2.36537	0.37121
C13	-0.74373	-2.33224	0.32013
C14	-0.71147	-2.26259	0.31165
C15	-0.51376	-2.11576	0.60230
C16	-0.44633	-2.08092	0.62620
C17	-0.40691	-2.04058	0.44370
C18	-0.43603	-2.03565	0.23922
C19	-0.50335	-2.07120	0.21422
N20	-0.75599	-2.01807	0.45900
N21	-0.74153	-2.43602	0.40691
N22	-0.33749	-2.00776	0.45359
C23	-0.79379	-2.47958	0.30090
C24	-0.82405	-2.55081	0.36087
C25	-0.86638	-2.59914	0.20320
C26	-0.89528	-2.66688	0.25769
C27	-0.88461	-2.68714	0.47545
C28	-0.84334	-2.63871	0.63497
C29	-0.81236	-2.57116	0.57689
C30	-0.92051	-2.75893	0.54032
C31	-0.88689	-2.79464	0.56697
C32	-0.92239	-2.86493	0.59851
C33	-0.99118	-2.89886	0.60954
C34	-1.02402	-2.86206	0.59738
C35	-0.98884	-2.79216	0.56452
C36	-0.81315	-2.75668	0.55972
C37	-0.88803	-2.90387	0.60783
C38	-1.02876	-2.97447	0.63091
C39	-1.09584	-2.89644	0.61160
C40	-1.02547	-2.75399	0.55425
C41	-1.12635	-2.89097	0.81207
C42	-1.19383	-2.92078	0.82065
C43	-1.23175	-2.95710	0.63004
C44	-1.20112	-2.96353	0.43089
C45	-1.13364	-2.93297	0.42127
C46	-0.89094	-2.93997	0.80776
C47	-0.85945	-2.97719	0.81421
C48	-0.82277	-2.97730	0.62370
C49	-0.81893	-2.94023	0.42479
C50	-0.85207	-2.90473	0.41588
C51	-1.30288	-2.98827	0.64197
C52	-0.78865	-3.01575	0.63625
H53	-0.68555	-2.16052	0.72828

H54	-0.74894	-2.10504	0.77573
H55	-0.68385	-2.01229	0.10307
H56	-0.62176	-2.06988	0.05497
H57	-0.55801	-2.23260	0.41455
H58	-0.61545	-2.35319	0.44320
H59	-0.79572	-2.35987	0.29562
H60	-0.73959	-2.23869	0.27507
H61	-0.54363	-2.14765	0.74209
H62	-0.42529	-2.08753	0.78327
H63	-0.40631	-2.00449	0.09809
H64	-0.52460	-2.06746	0.05321
H65	-0.81442	-2.46438	0.16232
H66	-0.87693	-2.58442	0.03767
H67	-0.92769	-2.70356	0.13330
H68	-0.83568	-2.65318	0.80549
H69	-0.78074	-2.53482	0.70354
H70	-0.79621	-2.73204	0.38810
H71	-0.79060	-2.78761	0.59124
H72	-0.79361	-2.71862	0.69882
H73	-1.02013	-2.98987	0.80325
H74	-1.01339	-2.99695	0.49215
H75	-1.08187	-2.99709	0.61533
H76	-1.05305	-2.76148	0.71727
H77	-1.05958	-2.77193	0.40492
H78	-0.99422	-2.69939	0.53732
H79	-1.09766	-2.86231	0.95942
H80	-1.21642	-2.91455	0.97453
H81	-1.22933	-2.99262	0.28354
H82	-1.11070	-2.93856	0.26722
H83	-0.91806	-2.93970	0.95824
H84	-0.86370	-3.00591	0.96830
H85	-0.79000	-2.93828	0.27672
H86	-0.84805	-2.87598	0.26217
H87	-1.32547	-2.99265	0.80927
H88	-0.79131	-3.04138	0.79640

Supplementary References

1. Feng, X., Ding, X. & Jiang, D. Covalent organic frameworks. *Chem. Soc. Rev.* **41,** 6010-6022 (2012).

2. Waller, P. J., Gándara, F. & Yaghi, O. M. Chemistry of covalent organic frameworks. *Acc. Chem. Res.* **48,** 3053-3063 (2015).

3. Zeng, Y., Zou, R. & Zhao, Y. Covalent organic frameworks for CO_2 capture. *Adv. Mater.* **28,** 2855-2873 (2016).

4. Sakaushi, K. & Antonietti, M. Carbon- and nitrogen-based organic frameworks. *Acc. Chem. Res.* **48,** 1591-1600 (2015).

5. Wan, S., Guo, J., Kim, J., Ihee, H. & Jiang, D. A belt-shaped, blue luminescent, and semiconducting covalent organic framework. *Angew. Chem. Int. Ed.* **47,** 8826-8830 (2008).

6. Yu, J.-T., Chen, Z., Sun, J., Huang, Z.-T. & Zheng, Q.-Y. Cyclotricatechylene based porous crystalline material: Synthesis and applications in gas storage. *J. Mater. Chem.* **22,** 5369-5373 (2012).

7. Jackson, K. T., Reich, T. E. & El-Kaderi, H. M. Targeted synthesis of a porous borazine-linked covalent organic framework. *Chem. Commun.* **48,** 8823-8825 (2012).

8. Côté, A. P. *et al*. Porous, crystalline, covalent Organic Frameworks. *Science*, **310,** 1166-1170 (2005).

9. Côté, A. P., El-Kaderi, H. M., Furukawa, H., Hunt, J. R. & Yaghi, O. M. Reticular synthesis of microporous and mesoporous 2D covalent organic frameworks. *J. Am. Chem. Soc.* **129,** 12914-12915 (2007).

10. Ding, S.-Y. *et al.* Construction of covalent organic framework for catalysis: Pd/COF-LZU1 in Suzuki–Miyaura coupling reaction. *J. Am. Chem. Soc.* **133,** 19816-19822 (2011).

11. Spitler, E. L. & Dichtel, W. R. Lewis acid-catalysed formation of two-dimensional phthalocyanine covalent organic frameworks. *Nat. Chem.* **2,** 672-677 (2010).

12. El-Kaderi, H. M. *et al*. Designed synthesis of 3D covalent organic frameworks. *Science* **316,** 268-272 (2007).

13. Hunt, J. R., Doonan, C. J., LeVangie, J. D., Côté, A. P. & Yaghi, O. M. Reticular synthesis of covalent organic borosilicate frameworks. *J. Am. Chem. Soc.* **130,** 11872−11873 (2008).

14. Wang, Z. *et al*. Phenanthro[9,10-d]imidazole as a new building block for blue light emitting materials. *J. Mater. Chem.* **21**, 5451-5456 (2011).

15. Xu, S.-Q. *et al.* The construction of a two-dimensional supramolecular organic framework with parallelogram pores and stepwise fluorescence enhancement. *Chem. Commun.* **51**, 16417-16420 (2015).
